# Towards patient-centered healthcare: a scoping review of soft skills training in medical schools

**DOI:** 10.1186/s12909-026-09059-0

**Published:** 2026-06-17

**Authors:** Daniela Mariana Zagalo, Cristiana Palmieri, Noelle Resende, Teresa Costa, Rute Dinis de Sousa, Helena Canhão, Fernando A. Bozza

**Affiliations:** 1 CHRC, LA-REAL, NOVA Medical School, Faculdade de Ciências Médicas, NMS, FCM, Universidade NOVA de Lisboa, Campo Mártires da Pátria, Lisbon, 1301169-056 Portugal; 2 NOVA Medical School, Universidade NOVA de Lisboa, Campo Mártires da Pátria, Lisbon, 1301169-056 Portugal; 3 CHRC, NOVA Medical School, Faculdade de Ciências Médicas, NMS, FCM, Universidade NOVA de Lisboa, Lisbon, Portugal; 4 The University of Sydney, Sydney, Australia; 5 Oswaldo Cruz Foundation, Rio de Janeiro, Brazil; 6 D’Or Institute for Research and Education, Rio de Janeiro, Brazil; 7 Episaúde - Associação Científica, Évora, Portugal

**Keywords:** Medical Education, Clinical Practice, Patient-Centered Healthcare, Interpersonal Skills, Cognitive Skills, Communication Skills, Emotional Intelligence, Humanism, Medical Ethics, Quality of Health Care

## Abstract

In response to the dynamic evolution of the medical profession, driven by digital transformation, demographic shifts, and global health challenges, medical schools are emphasizing the importance of soft skills alongside technical and digital expertise. This scoping review aimed to identify key concepts and core characteristics related to soft skills in medical literature. Following a comprehensive search across PubMed, Scopus, and Web of Science, supplemented by a manual search and conducted in accordance with PRISMA-ScR guidelines, 218 articles were identified. After screening and full-text evaluation, 83 studies met the inclusion criteria. The analysis of the literature revealed three recurring thematic axes: (1) the global trend of incorporating soft skills into medical education; (2) the adoption of innovative andragogical strategies to foster the development of these competencies; and (3) the persistent obstacles and limitations that hinder the effective implementation of soft skills training in medical curricula. The review identified critical research gaps and priority areas to strengthen the integration of soft skills-related competencies in medical curricula. These findings highlight the need for targeted strategies to develop compassionate, culturally responsive physicians capable of delivering high-quality, patient-centered care. By synthesizing current evidence and mapping key themes and limitations, this review provides a foundation for future educational interventions and policy development in soft skills training.

**Supplementary Information:**

The online version contains supplementary material available at 10.1186/s12909-026-09059-0.

## Background

The healthcare sector faces multifaceted challenges driven by digital transformation, demographic shifts, climate change, and persistent inequalities in healthcare access. The COVID-19 pandemic has accelerated some of these trends, reshaping healthcare delivery, education, and workforce dynamics [1–3]. Consequently, healthcare professionals must manage multiple uncertainties stemming from geopolitical conflicts, environmental crises, economic instability, migratory flows, and rapid technological advancement. Additionally, healthcare workers are frequently exposed to chronic stress, burnout, and psychological.

distress, highlighting the urgent need for resilient health systems and adaptive medical education [1–3].

In this evolving landscape, beyond technical and digital skills, soft skills have emerged as critical for effective healthcare practice. In this review, *soft skills* are defined as non-technical, transferable competencies that shape how physicians think, relate, and act in clinical practice [4]. They include intrapersonal capacities (e.g., self-awareness, emotional regulation, resilience/reflective practice) and interpersonal competencies (e.g., empathic communication, teamwork, leadership, conflict management), complementing technical (“hard”) skills [4]. The rapid transformations in social and working contexts compel medical education to develop innovative pathways that integrate technical, digital and soft skills with fundamental clinical expertise [5]. Despite various frameworks describing essential skills for the modern healthcare environment, including intrapersonal, interpersonal, social, emotional, and cultural competencies, there remains no consensus on terminology or precise definitions.

As a conceptual starting point for this scoping review, we adopt soft skills as an overarching category encompassing subjective and non-technical competencies. This skillset typically includes competencies from the intrapersonal domain, such as self-awareness, emotional regulation, stress management, and critical thinking, as well as the interpersonal domain, which encompasses, among others, empathy, communication, collaboration, and conflict resolution. Although terminology may vary across disciplines and contexts, there is growing convergence around the notion that soft skills involve the capacity to navigate complex social environments, manage emotions, collaborate effectively, and apply reflective and ethical reasoning [6–8]. These skills are generally distinguished from hard skills—that is, technical or domain-specific knowledge—and instead refer to cross-cutting competencies that support adaptability, interpersonal functioning, and personal agency [9].

Our conceptualization of soft skills draws on key frameworks, including the *World Economic Forum’s Education 4.0 Taxonomy (2023)* [10]; *the K4D report on 21 st Century Skills (2019)* [11]; and the *BESSI framework for social, emotional, and behavioral skills (Soto *et al*., 2022)* [12]. These studies synthesize a broad range of literature and frameworks developed by authors and organizations such as *Chalkiadaki (2018)* [13], *Vogt and Roblin (2012)* [14], *Scott (2015)* [15] and the *Organisation for Economic Co-operation and Development (OECD)* [16], offering a comprehensive foundation for defining, categorizing, and assessing soft skills in both educational and professional contexts.

Despite variations in terminology, these frameworks commonly identify three interrelated dimensions of soft skills: (1) a procedural dimension, related to how something is done (i.e., the skill or behavior itself); (2) an axiological dimension, which refers to the values and intentions that justify why a particular behavior is relevant or desirable; and (3) a technical dimension, which relates to knowledge associated with a particular context or discipline. While the designation and structure of these dimensions may vary across models, they provide a useful lens for interpreting the complexity of soft skills [10]. An illustrative synthesis of these dimensions is presented in Table 1 [10]. This multidimensional view helps distinguish soft skills from purely technical or disciplinary knowledge, while emphasizing their importance across domains. These competencies are increasingly valued for their role in enhancing social interaction, emotional regulation, ethical decision-making, and collaborative problem-solving [17].

**Table 1:** Ilustrative Conceptual Framework for Soft Skills Competencies [10]

Dimension	Description	Examples
Procedural	Related to *how* something is done, the skill or behavior itself	- Creativity, Critical Thinking, Digital Skills, Problem-Solving (cognitive domain) - Collaboration, Communication, Socio-emotional Skills (social/collective domain)
Axiological	Refers to the values and intentions that justify *why* a behavior is relevant or desirable	- Self-awareness, Grit, Initiative, Growth Mindset (self-regulatory) - Civic Responsibility, Empathy, Global, Citizenship, Environmental Concern (societal domain)
Technical	Relates to knowledge associated with a particular context or discipline	- Discipline-specific knowledge

Legend: Illustrative synthesis of soft skill dimensions—procedural, axiological, and cognitive—adapted from key conceptual frameworks, including the World Economic Forum’s Education 4.0: A Taxonomy for the Future of Learning (2023) [10]

Given their relevance for holistic development, multiple international initiatives—including *OECD’s Social and Emotional Skills Framework (2023)* and *UNESCO’s Futures of Education Report (2021)*—advocate for explicit integration of these competencies into educational systems, not only as learning outcomes but also as guiding principles for curriculum design and pedagogy [18, 19]. In this context, soft skills support critical thinking, value awareness, empathetic communication, and sensitivity to social, cultural, and economic diversity [18, 19]. For healthcare professionals and students, developing these competencies is essential to delivering ethical, effective, and compassionate responses to complex challenges [5, 20, 21].

Recent discussions in medical education and pedagogy have focused on establishing a shared terminology for soft skills in medicine, identifying core “humanistic competencies” essential for professional development, and defining the educational goals that innovative curricula should achieve to foster these “non-technical” skills [5, 21–24].

While the theoretical debate on teaching and cultivating soft skills in health professions is well established [5], their limited integration into medical training curricula highlights a significant gap. Academic institutions must take a proactive role in addressing current and future healthcare demands by evolving beyond knowledge delivery toward becoming catalysts of social transformation [21]. There is an urgent need to develop and implement andragogical strategies^1^ that comprehensively incorporate soft skills training into healthcare education, along with scalable and effective assessment methods to evaluate their impact on clinical outcomes [25].

To achieve these objectives, several core challenges must be addressed: the development and implementation of robust teaching methodologies for the transmission and practice of soft skills-related competencies; the creation of experimental environments to test and generate evidence on the effectiveness of training interventions; the formulation of standardized evaluation frameworks to measure learning outcomes and the impact of soft skills on clinical practice; and the integration of soft skills training as a cross-cutting, fundamental component of medical curricula [26–32].

In recent years, medical schools around the world have increasingly adopted innovative approaches to incorporate soft skills and social medicine competencies into their educational programs [32]. This shift reflects a growing recognition that effective healthcare delivery depends not only on technical expertise but also on emotional intelligence, communication, and a deep understanding of social contexts. Some examples are given in Table 2 [32]. Collectively, these initiatives represent a global movement that prioritizes not only clinical competence but also the development of compassionate, culturally sensitive, and socially responsible healthcare professionals.

**Table 2:** Examples of soft skills incorporation in the medical curriculum

Institution	Innovation	Source
Harvard Medical School	Incorporation of professionalism, communication abilities, and awareness of social determinants of health	[26]
Stanford Medical School	Educators-4-CARE (Compassion, Advocacy, Responsibility, and Empathy) program	[27]
Imperial College London	Integration of medical science with ethics, law, philosophy, history, and the arts	[28]
Mayo Clinic College of Medicine and Science	Teamwork, leadership, and communication through interdisciplinary collaboration	[29]
Ottawa University	Therapeutic and patient-centered communication, and ethical interactions	[30]
University of Calgary	Incorporation of social context into clinical teaching	[31]
University of Melbourne	Diagnostic reasoning, interpersonal communication, and clinical management through team-based learning strategies	[32]

Although there is broad recognition of the need for soft skills training, the literature reveals gaps in systematic, evidence-based strategies for their curricular integration. While various studies examine individual teaching methods, there is a lack of comprehensive synthesis identifying best practices, curricular frameworks, and assessment methodologies for embedding these skills in medical education. Additionally, limited research systematically evaluates the institutional and andragogical barriers to implementation [5, 7–9, 21, 24, 33–35].

This scoping review aims to identify and synthesize the key concepts, definitions, and terminologies related to soft skills as found in academic medical literature. By identifying, organizing, and analyzing existing evidence, the review seeks to support the integration of soft skills-related competencies into medical training. Special focus is given to innovative andragogical strategies, including narrative medicine, patient experience simulations, and other evidence-based educational interventions designed to enhance soft skills development. By clarifying the conceptual landscape and identifying core challenges, this study aims to provide a robust foundation for future andragogical and curricular innovations in the field.

## Methodology

This manuscript presents a comprehensive review of the current literature on soft skills within the context of medical education, with a specific focus on their importance in advancing patient-centered healthcare and improving clinical outcomes. The primary objective was to synthesize a clear understanding of the soft skills deemed relevant for inclusion in medical curricula, ultimately aiming to enhance medical training and its impact on clinical practice. To accomplish this goal, an exhaustive literature search was conducted in May 2024 across three major databases: PubMed, Scopus, and Web of Science. The search focused on studies discussing training, assessment, or integration of soft skills in medical schools. Only publications in English were considered, and no temporal restrictions were applied to ensure the inclusion of relevant studies regardless of publication date.

Databases were selected a priori to reflect the multidisciplinary nature of soft skills and their training within medical schools, in line with the scope of this scoping review on soft skills training and its contribution to patient-centered healthcare. We searched PubMed to capture the core biomedical and health sciences literature, given its strong coverage of medical journals and standardized indexing. To complement PubMed and reduce the risk of missing eligible studies due to database-specific coverage, we also searched Scopus and Web of Science, which provide broad multidisciplinary indexing and support retrieval of relevant educational and interdisciplinary records that may not be indexed in PubMed. Collectively, these databases were chosen to maximize the sensitivity and comprehensiveness of the search for studies reporting soft skills training in undergraduate medical education/medical schools and related outcomes.

The search strategy was implemented through the following query: ("soft skill*" OR "affective skill*" OR "interpersonal skill*" OR empathy OR compassion OR "emotional intelligence" OR "communication skill*") AND ("medical education" OR "medical training" OR "medical student") AND ("medical practice" OR "clinical practice" OR "clinical outcome") NOT Nursing.

The initial search yielded 192 articles: 26 from PubMed, 132 from Scopus, and 34 from Web of Science. In addition to the main database query, we conducted a supplementary manual search to ensure adequate capture of humanistic and professionalism-related concepts closely aligned with soft skills. Specifically, we used additional terms such as “Humanistic Medicine,” “Humanism Education for Medical Students,” “humanism in medicine,” and “medical ethics,” combined with core medical education and clinical practice terms. We also performed backward citation searching (snowballing) by reviewing the reference lists of included studies to identify potentially relevant records not retrieved through database indexing. Records identified through these supplementary approaches were incorporated into the screening workflow and assessed using the same eligibility criteria as database-derived records. Together, these supplementary methods identified 26 additional studies, bringing the total number of publications for further consideration to 218.

To manage and refine the selection process, Rayyan Professional software was used to eliminate duplicates [36]. This led to the removal of 65 duplicate entries, leaving a final set of 179 unique studies for screening. The screening was conducted by six independent reviewers, who first assessed the abstracts for alignment with the research objectives. To ensure objectivity, each reviewer was blinded to the decisions of others during the initial screening phase. Articles categorized as “uncertain” or “maybe” were discussed in consensus meetings. Consensus was achieved through structured group deliberation, and in cases of continued disagreement, a senior reviewer served as a tiebreaker to resolve conflicts and finalize decisions.

The exclusion of studies from the review was based on three criteria. First, articles that were classified as commentaries, letters, or opinion pieces were excluded, as they did not present original research or data that would contribute to answering the research question. Second, studies not published in English were excluded due to the limitations of available translation resources. Third, studies that were determined to be irrelevant to the central objectives of the review were excluded, such as those that did not directly address soft skills in medical education or did not provide relevant data pertinent to the research question.

**Fig. 1:**
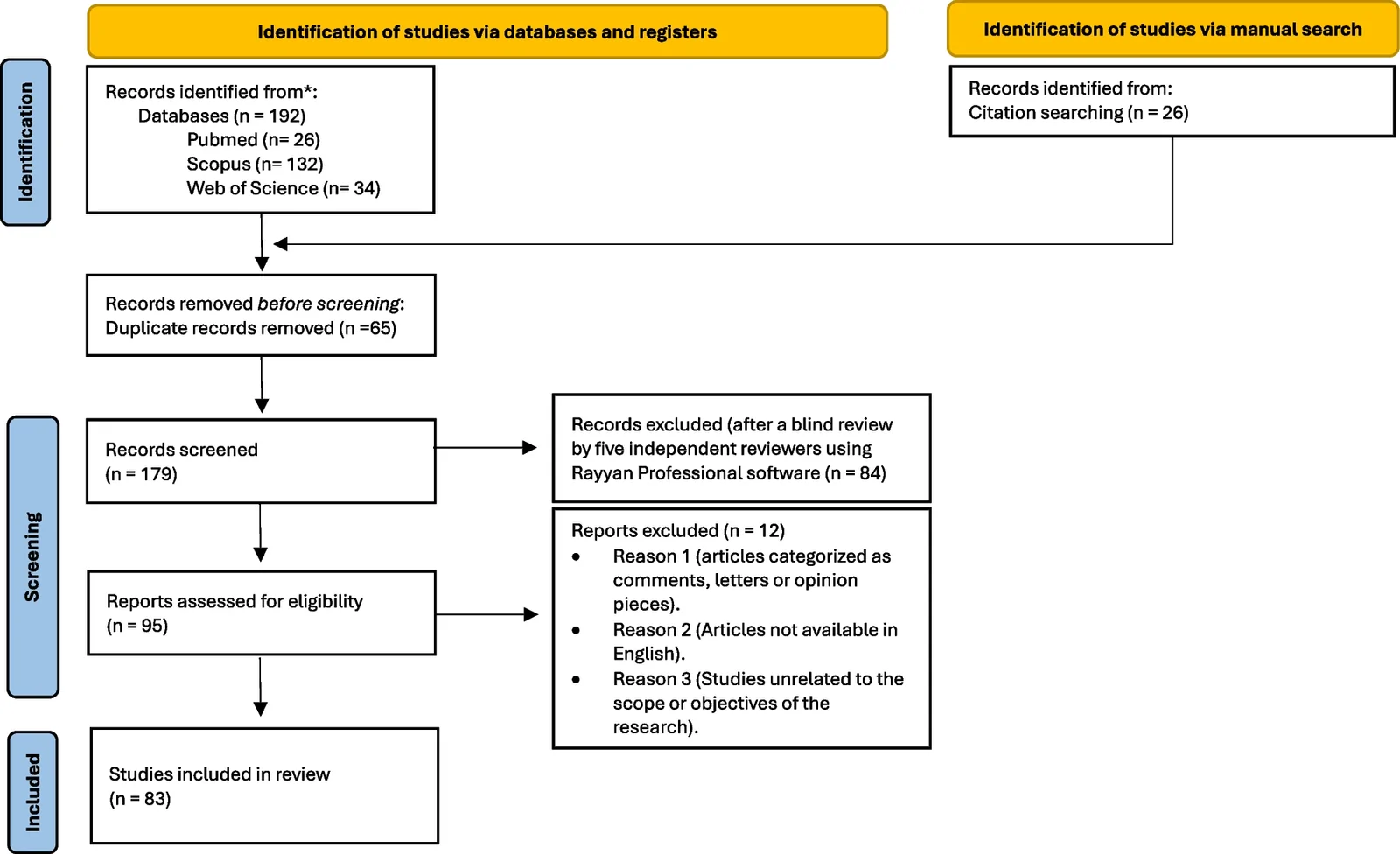
PRISMA flow diagram illustrating the electronic search strategy and the screening process for study selection [37]. Legend: Reason 1: Excluded due to publication type (articles categorized as commentaries, editorials, letters to the editor, or opinion pieces that did not present original research or empirical data); Reason 2: Excluded for language limitations (articles not published in English were omitted due to the absence of reliable translation resources); Reason 3: Excluded for lack of relevance (studies outside the scope of the review, particularly those not addressing soft skills within the context of medical education or failing to align with the conceptual objectives of the research)

Following full-text evaluation, 83 studies met the inclusion criteria and were incorporated into the final review. The entire literature search process adhered to the methodology outlined in the Preferred Reporting Items for Systematic Reviews and Meta-Analysis for Scoping Reviews (PRISMA-ScR) [37]. The PRISMA flow diagram, which illustrates the electronic search strategy, including both the query and reference list screening (snowballing), is presented in Fig. 1.

## Results

A total of 83 studies were included in the scoping review. The data extraction process was conducted systematically using a standardized Excel spreadsheet to ensure methodological consistency and data reliability. The extraction form was designed to capture detailed information from each study, including the study title, type of study (such as qualitative, quantitative, mixed-methods, randomized controlled trials, descriptive or systematic reviews), and the population under investigation. These populations varied widely and encompassed medical students, residents, physicians, or other interprofessional healthcare teams from diverse geographic and institutional settings. Additional fields included the main purpose of the study, the methodology employed, key findings, the authors’ conclusions, and complete bibliographic reference information. Within the included evidence base, empathy was the most frequently highlighted soft-skill focus (n = 22 studies), followed by humanism (n = 9). Communication skills were identified as a distinct sub-theme in n = 1 study (Table 3). These studies are detailed in Supplementary Materials (Appendix B) Table 5.

**Table 3:** Key findings from the scoping review

Theme	Sub-theme	Number of Articles Identified	References
Integration of soft skills into medical education	Empathy	22	[38–59]
	Communication Skills	1	[60]
	Humanism	9	[20, 22, 61–67]
Innovative teaching practices to embed soft skills	Art-Based Learning	2	[46, 68]
	Simulation-based learning	7	[40, 49, 51, 64, 69–71]
	Virtual Medical Communication (VMC)	10	[51, 54, 70, 72–78]
	Narrative medicine	6	[59, 67, 79–82]
	Design Thinking and Jigsaw Cooperative Learning	2	[83, 84]
	Reflective and Immersion-Based Learning	2	[85, 86]
Challenges and barriers	Time Constraints	2	[87, 88]
	Systemic barriers	1	[89]
	Emotional and Psychological Factors for Learners	3	[88, 90, 91]
	Adapting to Emerging Learning Modalities	2	[83, 92]

The scoping review highlights the integration of soft skills training as a burgeoning trend in medical education, reflecting the growing recognition among medical schools of the critical importance of cultivating compassionate, patient-centered care to meet the evolving demands of global healthcare. Three major themes have emerged from the analysis of the literature: a global shift towards the integration of soft skills into medical education, the use of innovative teaching practices to embed soft skills in medical education and the challenges and barriers to the effective implementation of soft skills training in medical education. Table 3 illustrate the main findings and related examples in the literature.

## Integration of soft skills into medical education

The integration of soft skills into medical curricula has become increasingly recognized as essential for developing well-rounded healthcare professionals [21]. The first outcome of the scoping review was identifying relevant soft skills for medical practitioners because of their role in fostering positive patient relationships and ensuring high-quality care. Embedding the teaching of these skills in the curriculum is crucial for preparing healthcare professionals to deliver compassionate and effective patient care [28, 93, 94]. The scoping review has identified three primary soft skills: empathy, communication, and humanism.

### Empathy

Empathy was addressed in n = 22 of the included studies, making it the most frequently highlighted soft-skill focus in this scoping review (Table 3). Clinical empathy, one of the most frequently discussed interpersonal capabilities that must be cultivated in medical education, is portrayed as a skill set encompassing interactional techniques and emotional regulation, to fulfil patient satisfaction expectations [48, 95]. This perspective aligns with empathy as a type of emotional labour [38], where healthcare providers manage their emotions to present a compassionate demeanour. Empathy is crucial to patient care, contributing to better patient outcomes and satisfaction [48]. Educational constructs aimed at teaching empathy and compassion have been explored, encompassing areas such as communication, mindfulness, early clinical exposure, technology-enhanced learning, and the use of arts and culture [93]. These approaches foster a deeper understanding of patient experiences and promote compassionate care among medical students. Interventional strategies, such as communication skill workshops, have shown promise in enhancing empathy among medical professionals.

### Communication Skills

Communication skills were identified as a distinct sub-theme in n = 1 included study (Table 3). Effective communication emerges from the review as pivotal in healthcare, directly influencing patient safety and care quality [96]. Training programs focusing on communication skills are essential for improving physician–patient interactions. These programs boost clinicians' confidence and lead to better patient outcomes. For instance, structured communication skills training (CST) programs have been shown to improve clinicians' abilities significantly. Psychiatrists who participated in CST demonstrated increased patient-centeredness and enhanced competence in using open-ended questions and social communication categories [39]. Another example to integrate communication skills into the teaching curriculum is structured modules focusing on attitudes, ethics, and communication, such as the Attitude, Ethics, and Communication (AETCOM) module [92]. This module has been well-received by medical students, with a significant majority of students reporting an improved understanding of sociocultural, ethical, and medico-legal aspects. Furthermore, panel discussions have been found to be an effective andragogical approach, and reflective exercises have been observed to contribute positively to students' learning experiences.

### Humanism

Humanism-related competencies were addressed in *n* = 9 studies (Table 3). Humanism, encapsulating virtues such as compassion, empathy, and respect, is critical to patient-centered care. Promoting humanism in medical education is crucial for fostering caring, trusting clinician-patient relationships, and the cultivation of qualities such as empathy, compassion, altruism, and respect for patients' dignity and beliefs. Educational approaches focused on humanistic competencies, such as reflective exercises, role-modelling, and community engagement, have demonstrated positive effects on students' attitudes and behaviours [97, 98]. Medical schools have implemented various strategies to embed humanistic values in their curricula, including initiatives that encourage students to engage with arts and humanities [20, 99]. These approaches aim to cultivate a deeper understanding of the human experience, empathy, and ethical reasoning, ultimately shaping the development of compassionate healthcare professionals, recognizing the importance of understanding the human experience in illness and recovery. By integrating humanistic values into medical training, educators seek to develop physicians who are not only clinically proficient but also attuned to the personal and emotional needs of their patients.

The analysis has also identified, alongside the above discussed soft skills, other skills that are relevant for preparing healthcare professionals to deliver compassionate and effective patient care (Table 4).

**Table 4:** Other key soft skills identified as essential for integration into medical education

Skills	Description	References
Active Listening	Engaging in clear, effective, and patient-centered conversations, including both verbal and nonverbal communication	[42, 100, 101]
Compassion	Understanding patient emotions, showing care, and maintaining a humanistic approach to medicine	[102–104]
Critical Thinking and Problem-Solving	Analyzing complex medical situations, making sound clinical judgments, and adapting to new challenges	[105–108]
Cultural Competence and Perspective-Taking	Understanding diverse patient backgrounds, ensuring inclusivity, and adapting care accordingly	[109–111]
Emotional Intelligence (EI) and Self-awareness	Recognizing, managing, and utilizing emotions effectively in clinical practice while reflecting on personal biases and behaviours	[33, 112–114]
Leadership and Mentorship	Guiding junior colleagues, fostering a culture of learning, and inspiring positive change in healthcare settings	[115–117]
Patient-Centered Care and Shared Decision-Making (SDM)	Engaging patients in their treatment choices, considering their values and preferences	[57, 118, 119]
Professionalism and Ethical Decision-Making	Upholding integrity, responsibility, patient autonomy, and ethical standards in medical practice	[120, 121]
Resilience and Coping Skills	Managing stress, preventing burnout, and maintaining mental well-being in a high-pressure environment	[122–124]
Teamwork and Collaboration	Working effectively within interdisciplinary healthcare teams, fostering mutual respect and cooperation	[34, 35, 125]

In summary, integrating soft skills training into medical education is essential for developing healthcare professionals who are not only technically proficient but also capable of delivering compassionate, patient-centered care. Emphasizing empathy, communication skills and humanism within medical curricula can lead to improved patient outcomes and satisfaction, as well as more fulfilling professional experiences for healthcare providers.

**Fig. 2:**
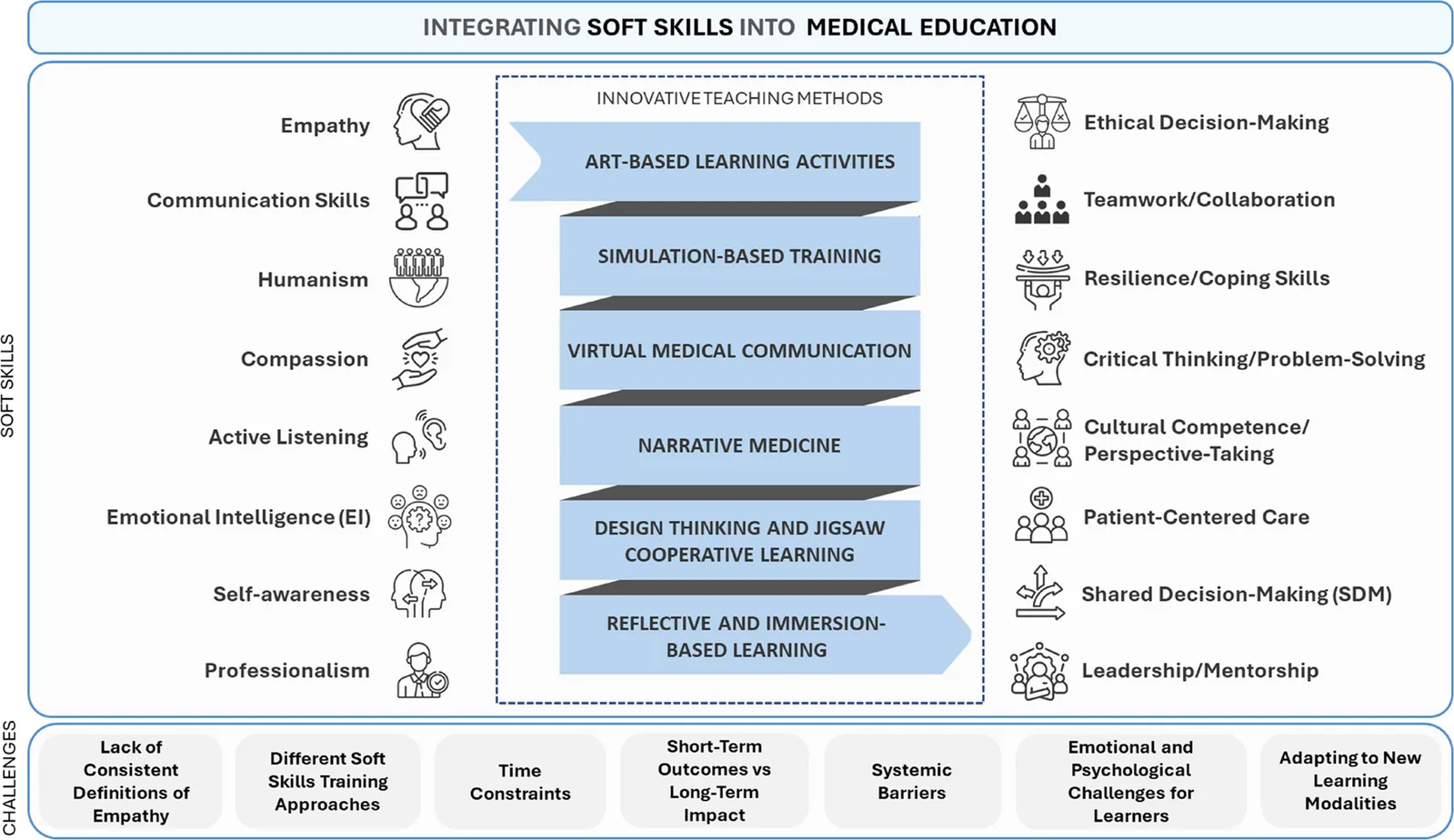
Integrating Soft Skills into Medical Education: Key Themes, Innovative Teaching Methods, and Challenges. Legend: This schematic synthesizes key soft skills relevant to patient-centered care, innovative andragogical strategies used to develop these competencies, and the principal challenges to their curricular integration identified in the scoping review. It highlights the alignment between intrapersonal and interpersonal competencies, educational approaches (e.g., simulation, narrative medicine, and reflective learning), and systemic, pedagogical, and learner-related barriers shaping soft skills training in medical education

Figure 2 presents a schematic summary integrating soft skills into medical education, highlighting innovative teaching methodologies and the key challenges associated with their integration.

## Innovative teaching practices to embed soft skills in medical education

In contemporary medical education, innovative teaching practices have been increasingly adopted to enhance the development of soft skills among students.

### Art-based learning activities

Experiential and art-based pedagogies, such as forum theatre, examining contemporary art, and utilizing comics-based curricula, have been shown to cultivate metacognition, emotional intelligence, and perspective-taking among students [41, 47, 68, 82]. This arts-integrated approach has been demonstrated to enhance observational and interpretive abilities, foster comfort with ambiguity, and promote collaborative critical thinking. By engaging with artistic mediums, students develop a more nuanced understanding of patient experiences and learn to appreciate multiple viewpoints in healthcare delivery.

### Simulation-based training

This method engages students in role-play and standardized patient encounters to practice communication, shared decision-making, and emotional regulation in a controlled, low-risk environment [126–131]. Through simulated interactions, students can practice and refine their communication skills in a controlled environment, receiving feedback that is essential for their professional growth. These initiatives provide trainees with valuable insights into the experiences of patients and their families, emphasizing the importance of effective communication, responsiveness, and conscientious care.

### Virtual Medical Communication

This approach utilizes digital technologies such as virtual reality and avatar-based interactions, has emerged as a promising approach in medical education to foster soft skills [77, 83, 132–134]. These innovative techniques allow medical students to develop soft skills-related competencies while navigating the challenges of remote healthcare delivery, a critical skill set for the evolving healthcare landscape. The incorporation of virtual human technology into simulation-based training programs has demonstrated the ability to improve interdisciplinary teamwork and communication among medical professionals [135]. Incorporating interactions with simulated patients and colleagues, students can practice essential skills, such as assertive communication, empathy, and collaborative problem-solving, in a safe and controlled environment.

### Narrative medicine

Narrative medicine, which involves the use of reflective writing, literature, patient narratives and storytelling, is another approach that has demonstrated significant benefits in medical education [59, 67, 79, 81, 82, 136, 137]. Engaging with patients' stories allows students to develop a deeper understanding of patient experiences, fostering a humanistic view of illness and strengthening the doctor-patient relationship. Narrative practices have shown not only to enhance residents' professional growth and communication skills but also to foster empathetic and effective care [58, 136, 138].

### Design Thinking and Jigsaw Cooperative Learning

These methodologies have shown to significantly improve collaboration, problem-solving, and critical thinking through structured group learning activities where students take responsibility for different aspects of a case and teach one another [84]. Both quantitative and qualitative analyses support these findings, highlighting the method's effectiveness in medical education and indicating that such methods foster a collaborative learning environment that enhances these essential competencies [83, 84].

### Reflective and Immersion-Based Learning

This approach is focused on the collection of student reflections. This practice encourages students to critically assess their experiences, leading to improved understanding and application of communication skills, ethical considerations, and sociocultural attitudes in clinical practice [85, 138]. These are immersive experiences to help students process their clinical encounters, develop emotional intelligence, and reinforce professionalism.

In summary, contemporary medical education employs innovative teaching practices to enhance students' soft skills. These include, among others, integrating contemporary art to improve observational and critical thinking abilities, utilizing patient experience simulations to emphasize effective communication, adopting narrative medicine to foster empathy and strengthen doctor-patient relationships, and incorporating reflective learning to encourage students to critically assess their experiences and develop emotional intelligence.

## Challenges and barriers

The analysis of the studies has identified some challenges in relation to the integration of soft skills into medical education.

The literature review demonstrated a significant lack of consistent definitions of empathy, leading to uncertainty about which dimensions of this construct should be addressed [52, 53]. While training programs aimed at improving empathy have demonstrated significant enhancement in students' abilities to communicate empathetically [139–142], many studies have failed to provide clear and consistent definitions of empathy [143–145]. This inconsistency limits the validity and usefulness of the research, indicating a need for more comprehensive and qualitative approaches.

The analysis of the bibliographic corpus has also identified that communication skills training programs can foster students’ skills and cultivate their ability to communicate effectively. For example, a three-day program aimed at enhancing residents' patient-centered communication and empathy skills demonstrated positive results [60]. Randomized controlled trials have reported measurable benefits, such as increased empathy and targeted communication skills [39]. However, the variable approaches to communication skills training make it challenging to evaluate their effectiveness. While the existing research on communication skills training programs has demonstrated positive short-term outcomes, the long-term impact on skill retention and actual clinical performance remains an area that requires further investigation to validate their significance in medical education [40, 60]. Moreover, further validation studies examining specific models and frameworks would strengthen the evidence for communication skills training [39], and larger studies utilizing valid measurement methods are needed to better understand the experiences and feelings of both physicians and patients [48].

The analysis has also identified, alongside challenges specifically related to empathy and communication skills, obstacles and constraints in teaching soft skills in medical education:

*Time Constraints*: Heavy clinical and academic workload leave limited time in packed medical curricula to the development of soft skills [87, 88].

*Systemic barriers*: Institutions may lack the necessary culture, policies, or incentives to integrate soft skills training effectively [89].

*Emotional and psychological challenges for learners*: Some students struggle with the emotional demands of difficult conversations (e.g., breaking bad news, dealing with suffering patients). Without proper support, students may hesitate to engage in vulnerable discussions about emotions and biases [88, 90, 91].

*Challenges in adapting to new learning modalities*: Telemedicine and virtual care require new skill sets that are often overlooked in traditional training [83, 92].

## Discussion

Integrating soft skills training into medical education is widely recognized as essential for fostering compassionate, patient-centered care—an essential element for addressing global healthcare needs [46, 51, 146]. Our review highlights a growing trend in medical schools toward embedding these competencies within curricula through diverse educational interventions (Fig. 2). Approaches range from communication skills training programs to the implementation of multifaceted educational interventions focused on enhancing doctor-patient relationships [60]. Despite progress, effectively integrating these skills remains challenging, highlighting the need for ongoing innovation in teaching methods, along with further research to evaluate the long-term effects of such interventions [48, 87, 147].

Evidence from the literature consistently supports the impact of brief, targeted interventions on improving empathy, compassion, and communication skills among medical students [46, 48]. Modalities such as simulation-based learning, narrative medicine, and reflective writing have significantly enhanced these interpersonal qualities [58, 85, 126–131, 136, 138, 138]. These approaches foster emotional intelligence [33, 112–114] and offer opportunities for students to engage with real-world clinical scenarios, thus contributing to the development of both cognitive and affective dimensions of communication [63, 77]. Furthermore, the cultivation of humanism has been identified as a crucial component of medical education [20, 22, 66]. The integration of humanistic values, such as empathy, compassion, and respect for patient dignity, is crucial for preparing well-rounded professionals capable of delivering patient-centered care [63, 64, 148]. The emphasis on humanism in medical education reflects a broader shift towards a more balanced and holistic approach to healthcare, where technical expertise is complemented by soft skills and a commitment to the well-being of both patients and providers [20, 62, 64].

Although several interventions have demonstrated short-term improvements in empathy and communication skills among medical students, the evidence regarding their persistence over time remains inconsistent [39, 56, 67, 79]. Most studies rely on self-reported outcomes, small samples, or cross-sectional designs, which limits the ability to infer sustained behavioral change in clinical contexts. Recent longitudinal investigations confirm this concern: Baugh et al. (2020) found little evidence of lasting empathy enhancement beyond training periods [149], while Kötter et al. (2021) and Ardenghi et al. (2024) reported fluctuating empathy trajectories throughout medical education, influenced by curriculum structure, stress, and exposure to clinical environments [150, 151]. Carrard et al. (2025) further demonstrated that while cognitive and affective empathy may increase during early training, behavioral empathy tends to plateau later, highlighting a persistent gap between knowing, feeling, and acting empathically in professional settings [152]. From a conceptual standpoint, the development of empathy and relational competence is a dynamic, context-dependent process shaped by individual reflection, institutional culture, and the “hidden curriculum” of medical training. Future longitudinal and mixed-method studies (integrating behavioral observation, patient-reported outcomes, and reflective assessments) are essential to capture the evolution of soft skills from education into clinical practice and to inform institutional strategies that foster sustainable, compassionate, patient-centered care [70, 72, 153]. Such research could also help determine how soft skills influence patient outcomes, healthcare delivery, and provider well-being over time, which would provide a more comprehensive understanding of the effectiveness of these interventions.

Another emerging area of focus is interprofessional education (IPE), which has proven instrumental in fostering collaboration across healthcare disciplines [154, 155]. By providing students with the opportunity to work alongside nurses, social workers, and allied health professionals, IPE enhances critical teamwork, communication, and leadership skills. These competencies are crucial in delivering high-quality, patient-centered care in today’s complex healthcare environment, where interdisciplinary collaboration is paramount. Integrating IPE more thoroughly into medical curricula ensures that students are well-prepared for the collaborative nature of modern clinical practice, appreciating the value of diverse perspectives in patient care [154, 155].

Equally important is the integration of medical ethics and professionalism within soft skills training [22, 66, 93, 120, 120, 156–158]. Ethical principles, such as respect for patient autonomy, trust, and transparency, are fundamental to effective communication in healthcare. Medical ethics should not be taught in isolation but integrated into the soft skills curriculum, reinforcing the importance of professionalism in the delivery of compassionate, patient-centered care [121, 122].

Despite growing recognition of the importance of soft skills in medical education, significant gaps remain. One critical area is the intersection between technological advancements and the development of soft skills. As digital tools, artificial intelligence, and telemedicine increasingly shape clinical practice, there is a pressing need to understand how these shifts impact the cultivation and application of interpersonal competencies such as empathy, communication, and ethical reasoning [159–161]. Additionally, there is a lack of robust methodologies to evaluate the long-term effectiveness of soft skills training, particularly regarding its impact on clinical decision-making, patient satisfaction, and health outcomes [162], and a few studies only have examined the long-term impact of soft skills interventions [163, 164]. These gaps call for research into teaching models that combine tech fluency with soft skills and for scalable, validated assessment tools. Emerging work highlights the urgency of aligning soft skills education with the evolving technological and systemic contexts of modern medicine [108, 157, 165–167].

## Limitations

While the literature provides valuable insights into the integration of soft skills in medical education, several limitations must be acknowledged.

A limitation of this scoping review is that the literature search was conducted in three databases (PubMed, Scopus, and Web of Science); therefore, studies indexed exclusively in other relevant databases (e.g., discipline-specific education or psychology databases) may not have been retrieved. Although we supplemented database searching with backward citation searching (snowballing) to improve completeness, it is still possible that some relevant evidence was not captured. A predominant reliance on studies published in English presents a linguistic and cultural bias that may overlook important andragogical innovations and contextual nuances from non-English-speaking regions. This limitation constrains the generalizability of findings, especially given the cultural variability in how empathy, communication, and professionalism are taught, perceived, and enacted across global healthcare systems.

Many studies were conducted in high-income Western countries and often employed different outcome measures, with short-term evaluations predominating. This heterogeneity makes cross-study comparisons difficult and limits broader applicability of the findings. Additionally, the lack of standardized definitions and validated assessment tools for soft skills, coupled with the absence of formal methodological quality appraisal, restricts the ability to evaluate the rigor and reliability of existing evidence.

Finally, there is a lack of longitudinal studies examining the sustained impact of soft skills training throughout medical careers, which further limits our understanding of long-term effectiveness. Addressing these gaps requires not only more inclusive, globally representative studies, but also a concerted effort to develop culturally sensitive, validated methodologies that can meaningfully assess the longitudinal impact of soft skills training across diverse educational and healthcare contexts.

## Conclusions

This scoping review underscores the critical importance of integrating soft skills into medical education, emphasizing a growing consensus on their value in shaping compassionate, patient-centered healthcare professionals. As healthcare systems face increasingly complex challenges, the incorporation of competencies such as empathy, communication, professionalism, and emotional intelligence has never been more essential. Skills like active listening, self-awareness, and ethical decision-making are key to fostering effective patient-provider relationships and ensuring high-quality care. Furthermore, competencies in teamwork, resilience, critical thinking, and cultural competence are essential in addressing the diverse demands of modern healthcare. Patient-centered approaches, such as shared decision-making and leadership, further enhance the effectiveness and compassion of healthcare delivery. These multifaceted skills are critical for improving both patient outcomes and the well-being of healthcare professionals.

Despite considerable progress in embedding soft skills into curricula, significant barriers remain, particularly in terms of consistency, effective implementation, and the development of evidence-based frameworks for assessing their impact on clinical outcomes. Challenges such as institutional constraints and the need for scalable assessment tools highlight the necessity of a more systematic, comprehensive approach to teaching non-technical skills in medical education.

Looking ahead, future research must focus on longitudinal studies that evaluate the long-term effects of soft skills interventions on healthcare delivery and patient outcomes. There is an urgent need for the development of standardized assessment tools that capture the nuanced impact of these skills on clinical performance and patient satisfaction. Additionally, addressing institutional and andragogical barriers is critical to ensuring that soft skills training becomes a core, integrated component of medical curricula worldwide.

Based on the findings from the literature and institutional case studies, we propose the following recommendations to further enhance the integration of soft skills training into medical education:

- Adopt a competency-based, holistic approach to curriculum design that prioritizes the development of soft skills alongside clinical expertise. This approach should integrate a variety of teaching methods, such as role-playing, simulated patient encounters, and small-group discussions, to foster empathy and communication skills. Clinical experiences should provide ample opportunities for students to engage in compassionate patient interactions, with faculty support and feedback to help refine these skills.
- Emphasize patient-centered care by ensuring that students learn the importance of active listening, empathy, and patient involvement in healthcare decisions. This can be achieved through coursework and clinical experiences that highlight the significance of treating patients as individuals with unique needs, preferences, and values. Encouraging shared decision-making and involving patients in their care planning can strengthen students’ understanding of the human aspect of healthcare.
- Incorporate interprofessional education more thoroughly into medical curricula. By engaging in collaborative learning with other healthcare professionals, students will develop critical teamwork, communication, and leadership skills, all of which are essential for providing comprehensive, patient-centered care.
- Integrate medical ethics and professionalism into the soft skills training curriculum, ensuring that students develop an understanding of the ethical principles that underpin doctor-patient relationships. Trust, transparency, and mutual respect should be highlighted as key elements of effective communication and patient care.
- Provide opportunities for continuous reflection, feedback, and self-assessment throughout medical education and professional development. Students should be encouraged to reflect on their communication practices regularly and to seek feedback from patients, peers, and faculty to refine their interpersonal skills. This process of ongoing learning and self-improvement will promote the long-term development of compassionate and effective healthcare professionals.

To translate these insights into tangible improvements, it is imperative that key stakeholders take coordinated action. Universities and medical educators must actively champion the integration of soft skills within curricula, ensuring these competencies receive equal emphasis alongside clinical training. Higher education governing bodies and accreditation agencies should establish clear standards and guidelines that mandate the inclusion and rigorous assessment of soft skills in medical programs. Additionally, curriculum developers are encouraged to design innovative, evidence-based methodological strategies and scalable evaluation tools that address the unique challenges of teaching soft skills. By collectively committing to these efforts, stakeholders can drive systemic change that equips future healthcare professionals with the comprehensive skill set needed to excel in increasingly complex clinical environments and deliver patient-centered care.

To operationalize these efforts, medical schools could allocate approximately 10–15% of curriculum hours to structured soft skills training, progressively distributed throughout pre-clinical and clinical phases to ensure longitudinal reinforcement. Curriculum committees may introduce mandatory modules on communication, empathy, teamwork, and ethical decision-making early in medical education, with continued emphasis during clinical rotations. Incorporating formative and summative assessments—such as Objective Structured Clinical Examination (OSCE) stations, reflective portfolios, and multi-source feedback—is essential to monitor progress and ensure accountability.

Furthermore, collaboration with accreditation bodies to define minimum competency standards for non-technical skills can help solidify their institutionalization. Higher Education governing bodies should also provide guidelines to gradually implement these frameworks over a three-to-five-year period, allowing time for adaptation, faculty capacity building, and evaluation of results. Prioritizing faculty development initiatives will be critical to equip educators with evidence-based tools for effectively teaching and assessing soft skills competencies.

This review substantially contributes to the growing body of literature on soft skills in medical education and offers a structured roadmap for future research and policy development. It calls on medical schools, educators, and policymakers to prioritize soft skills-related competencies alongside clinical expertise in preparing healthcare professionals to navigate the complexities of contemporary medicine. By fostering a more holistic approach to medical education, we can ensure that the next generation of healthcare professionals is not only technically proficient but also deeply committed to delivering compassionate, patient-centered care.

## Supplementary Information

Supplementary Material 1. [20, 22, 29, 34–88, 92, 93, 108, 110, 118, 146, 147, 152, 155, 157, 168–182]. 

## Data Availability

All data generated or analyzed during this study are included in this published article [and its supplementary information files].

## Abbreviations

ACGME: Accreditation Council for Graduate Medical Education
AETCOM: Attitude, Ethics, and Communication Module
ACP: American College of Physicians
BBN: Breaking Bad News
CA-1: Clinical Anesthesia Year 1 residents
CINAHL: Cumulative Index to Nursing and Allied Health
COMLEX-USA: Comprehensive Osteopathic Medical Licensing Examination of the United States
CPX: Clinical Performance Examination
CST: Communication Skills Training
E4C: Educators-4-CARE [Compassion, Advocacy, Responsibility, and Empathy] program
EM: Emergency Medicine
EI: Emotional Intelligence
EO: External Observers
EPHRC: Ethics, Professionalism and Human Rights Committee
FT: Forum Theater
FGDs: Focus Group Discussions
GLM: General Linear Model
GPAT: Global Patient Assessment Tool
HEARS: Human Emotion and Response in Surgery
HET: High Empathic Tendency
IICs: Intra- and Interpersonal Competences
IRI: Interpersonal Reactivity Index
JSPE: Jefferson Scale of Physician Empathy
JSE-S: Jefferson Scale of Empathy—Student Version
JFSE: Jefferson Scale of Physician Empathy
LET: Low Empathic Tendency
OSCE: Objective Structured Clinical Examination
PERCS: Program to Enhance Relational and Communication Skills
PCSS: Patient-Centered Score Sheet
PRISMA-ScR: Preferred Reporting Items for Systematic Reviews and Meta-Analysis Statement for Scoping Reviews
RCT: Randomized Controlled Trial
RIAS: Roter Interaction Analysis System
SDM: Shared Decision-Making
SPIKES: A communication protocol used to deliver bad news (Setting, Perception, Invitation, Knowledge, Empathy, and Summary)
SP: Standardized Patient
SFM: Standardized Family Member
TMMS: Trait Meta-Mood Scale
USMLE: United States Medical Licensing Examination
VCE: Virtual Clinical Encounter
VOSviewer: Software used for visualizing bibliometric networks
VR: Virtual Reality
VMC: Video-Mediated Communication

## Footnotes

1 *Pedagogy* refers to the methods and practices of teaching children, focusing primarily on their specific learning needs. In contrast, *andragogy* refers to the methods and practices of teaching adults, emphasizing self-direction, experience-based learning, and practical relevance. Accordingly, we use the term *andragogical* throughout this article whenever it is considered appropriate.
