# Supplementary material for: Towards patient-centered healthcare: a scoping review of soft skills training in medical schools
Source: BMC Med Educ. 2026 Jun 17;26:1379. doi: 10.1186/s12909-026-09059-0 (PMC13508354; doi:10.1186/s12909-026-09059-0)
Supplement: Supplementary file 1 [file 12909_2026_9059_MOESM1_ESM.docx]

**Supplementary Material**

**Appendix A**

**Electronic Database Search Strategy**

The electronic database search was conducted in May 2024 across PubMed, Scopus, and Web of Science. The search aimed to capture a comprehensive set of studies addressing soft skills within medical education. No date limits were applied, and only English-language articles were considered. Boolean operators and truncation symbols were used to optimize sensitivity and specificity of search results.

**Databases Searched:**

PubMed

Scopus

Web of Science

**Search Query Applied Across All Databases:**

("soft skill*" **OR** "affective skill*" **OR** "interpersonal skill*" **OR** empathy **OR** compassion **OR** "emotional intelligence" **OR** "communication skill*")

**AND**

("medical education" OR "medical training" OR "medical student")

**AND**

("medical practice" **OR** "clinical practice" **OR** "clinical outcome")

**NOT**

Nursing

**Manual Supplementary Search Strategy**

A manual supplementary search was also conducted to capture studies emphasizing the humanistic aspects of medical education that may not have been retrieved in the initial query. The following keywords were searched in combinations using Boolean logic:

("medical education" **OR** "medical training" **OR** "medical student")

**AND**

("medical practice" **OR** "clinical practice" **OR** "clinical outcome")

**AND**

("humanistic medicine")

**Appendix B**

Table 1. Results of the bibliographic corpus analysis.

| **Nº** | **Title** | **Type of Study** | **Population** | **Main Purpose** | **Methodology** | **Key Results** | **Conclusions** | **Reference** |
| --- | --- | --- | --- | --- | --- | --- | --- | --- |
| **1** | Clinical empathy as emotional labor in medical work. | Qualitative Study. | Medical students and residents from two United States medical schools. | To examine how clinical empathy is operationalized, taught, and used as emotional labor in contemporary US medical education. | Case study Interpretive analysis of interviews with and ethnographic observations. | Clinical empathy is taught as interactional and emotion management skills to meet patient satisfaction expectations. (2) Emotional labor is a strategic tool in clinical practice, shaped by consumerism and corporatization. (3) Touch is included as a culturally variable element of clinical empathy. | Clinical empathy evolved into a standardized, taught performance that reflects broader sociocultural and economic pressures, with implications for physician autonomy, burnout, and global medical education practices. | [34] |
| **2** | Communication skills in the training of psychiatrists: A systematic review of current approaches. | Systematic Review. | Psychiatric trainees and clinicians participating in communication skills training (CST) programs. | To examine the outcomes of communication skills training interventions in psychiatric specialty training. | Systematic review of 12 studies (identified in a corpus of 2574 English-language published articles the efficacy of communication skills training. | CST improved communication skills, clinician empathy, and psychotherapeutic interviewing skills. (2) Most studies were non-randomized, small-scale, and lacked long-term outcomes on patient care or skill retention. (3) randomized controlled trials showed measurable benefits, such as empathy improvement and targeted skills enhancement. | The heterogeneity of communication skills training is a barrier to evaluating the efficacy of different communication skills training programmes. More rigorous research is needed to establish efficacy, long-term retention, and patient outcome impact | [35] |
| **3** | How to become an excellent pediatric resident: a qualitative comparative study from China. | Qualitative Comparative Study. | 23 pediatric residents at a children’s hospital in Beijing. | To explore the competency configurations of excellent pediatric residents and general pediatric residents and to provide a scientific reference for the talent training and career development of pediatric residents. | Behavioural event interviews’ data was coded to summarize the competency of pediatric residents. Performance-score: based on various aspects of competency; crisp-set qualitative comparative analysis method. | Identified six core competencies for pediatric residents: professional spirit, clinical skills, communication, learning ability, mental capacity, and research ability. (2) Four competency combinations for excellence: clinical type, research type, all-around development type, and high emotional intelligence type. Three competency deficiencies for general performance: lack of mental capacity, professional spirit, or communication ability. | Excellent pediatric residents should specialize in clinical practice, scientific research or communication skills. | [155] |
| **4** | Emotional intelligence in medicine: a systematic review through the context of the ACGME competencies. | Systematic Review. | Clinicians, medical students and medical school applicants. | To evaluate evidence for Emotional intelligence (EI) in medicine through the context of the Accreditation Council for Graduate Medical Education (ACGME) competencies. | Systematic literature search of databases and grey literature for empirical studies on EI in medicine from 1980–2009. 16 articles were included. | EI correlates positively with competencies like empathy, teamwork, communication, stress management, leadership, and doctor-patient relationships. Women tend to have higher EI than men (six of seven studies). | EI is relevant to many ACGME competencies and can enhance medical education and clinical outcomes; further research is required. | [108] |
| **5** | Assessing interpersonal and communication skills in radiation oncology residents: a pilot standardized patient program. | Mixed method Pilot Study. | 11 radiation oncology residents paired with 6 faculty members, using standardized Patients (Trained individuals role-playing patient scenarios). | Design of a novel, pilot standardized patient (SP) program focused on improving communication skills of radiation oncology residents, particularly in delivering bad news. | Two case scenarios: one regarding treatment failure; other regarding change in treatment plan.  Each encounter was scored by the SPs, observing faculty, and residents themselves based on the Kalamazoo guidelines. | Resident performance ratings were “good” to “excellent”. Faculty scored residents higher than residents scored themselves; SPs' ratings were intermediate. Poor inter-rater agreement highlighted differing perceptions among evaluators. SP feedback identified areas for improvement (less jargon, increasing empathy, and encouraging collaborative decision-making). | The program was well received by residents and faculty and regarded as a valuable educational experience that could be used as an annual feedback tool. | [65] |
| **6** | "We're All Time Poor": Experienced Clinicians' and Students' Perceptions of Challenges Related to Patient Communication. | Qualitative Study. | 20 medical students and 19 experienced clinicians from a range of medical disciplines. | Exploration of the patient-communication challenges faced by both medical students and experienced clinicians. | Convenience and snowball sampling; semi-structured qualitative interviews coded for challenges in medical communication; participants included students across program years and clinicians from varied specialties. | Common challenges include time constraints, rapport building, patient reluctance, omissions, and biases. Junior students report greater concerns about rapport and difficult topics, while clinicians note challenges like implicit bias and premature closure. | Communication skills training should address specific challenges, use experiential learning methods, and provide tailored feedback to enhance both student and clinician capabilities over time. | [83] |
| **7** | From contemporary art to core clinical skills: observation, interpretation, and meaning making in a complex environment. | Qualitative Study. | Medical students at Keck School of Medicine of the University of Southern California. | To enhance clinical skills through observation, description, and interpretation of contemporary art. | Guided instruction using nonrepresentational contemporary art; group discussions; reflective sessions. | Students improved observational and interpretive skills, learned to embrace ambiguity, and applied collaborative critical thinking. | Engaging with contemporary art fosters metacognitive skills, teamwork, and processes parallel to effective clinical practice. | [64] |
| **8** | Enduring impact of communication skills training: results of a 12-month follow-up. | Quantitative, randomized controlled trial. | 160 oncologists from 34 cancer centers in the UK. | To evaluate the long-term effects of a communication skills training program on oncologists' clinical practice. | Randomized 2x2 factorial design; videotaped consultations at baseline and 3- and 12-month follow-ups; robust Poisson analysis. | Skills like focused questioning and summarizing persisted or improved, but empathy expressions declined; no increase in consultation length. | Communication skills training has a lasting impact on oncologists’ practice, with some skills emerging over time; empathy needs reinforcement. | [68] |
| **9** | Intern as Patient: A Patient Experience Simulation to Cultivate Empathy in Emergency Medicine Residents. | Qualitative descriptive study. | Emergency medicine interns (8 of 10 interns participated) from a single academic center. | To evaluate whether a patient experience simulation during EM residency orientation helps develop empathy in interns. | Interns participated as patients or family members in an ED simulation, followed by focus groups analyzed using grounded theory. | Interns gained insights into patient/family experiences, emphasizing communication, responsiveness, and conscientious care; all valued the simulation. | Patients experience simulations are feasible and effective tools for fostering empathy; further research on long-term effects is needed. | [36] |
| **10** | "Brought to life through imagery" - animated graphic novels to promote empathic, patient-centered care in postgraduate medical learners. | Qualitative descriptive study. | Fourth- and fifth-year endocrinology residents at the University of Toronto (2017 cohort). | To assess factors associated with empathy decline and evaluate a comics-based curriculum for enhancing empathy and wellness. | Participants completed a 12-month course using graphic novels, followed by post-course interviews analyzed with constant-comparison methods. | Residents valued the curriculum for addressing real-world challenges, improving empathy, and promoting resilience; feedback was mixed. | Comics-based curricula can enhance empathy and reduce burnout in medical residents, though optimizing for diverse learners remains challenging. | [37] |
| **11** | Teaching counselling skills to fourth-year medical students: a dilemma concerning goals. | Quantitative, evaluative study. | Fourth-year medical students (N=63), University of Adelaide. | To evaluate the impact of General Practice training on students' communication skills, specifically empathy. | Students completed an Empathy Rating Scale before and after a 15-hour General Practice training segment. | No increase in empathetic responses; students continued to offer advice or closed questions rather than empathy. | The study suggests insufficient training time and highlights a conflict in medical education between problem-solving and patient-centered care. | [38] |
| **12** | Direct observation for assessing emergency medicine core competencies: interpersonal skills. | Quantitative, secondary data analysis. | Emergency medicine residents (1993–2000), MetroHealth Medical Center and Cleveland Clinic Foundation. | To assess residents' interpersonal skills using direct observation in emergency medicine. | Secondary analysis of direct observation evaluation forms completed for residency classes from 1993–2000. | Direct observation was not effective for identifying interpersonal skills issues; standardization may reveal differences among residents. | Direct observation has potential for assessing interpersonal skills but requires further validation and standardization. | [181] |
| **13** | A holistic approach of personality traits in medical students: An integrative review. | Qualitative, narrative overview. | Medical students, physicians, healthcare professionals. | To highlight the impact of personality traits on medical performance, communication, and patient outcomes. | Narrative review of literature on personality, empathy, emotional intelligence, and communication in medical practice. | Personality traits influence medical specialty choice, communication, empathy, and interprofessional collaboration, impacting both physician satisfaction and patient care. | Understanding personality traits is crucial for medical training, enhancing communication skills and improving patient outcomes. | [182] |
| **14** | Applying empathic communication skills in clinical practice: medical students’ experiences. | Qualitative Descriptive Study. | 18 undergraduate medical students at Stellenbosch University, South Africa. | To explore students’ perceptions of applying empathic communication skills in clinical practice. | Focus-group discussions; thematic analysis of transcribed recordings. | Students valued skills learned but faced challenges like emotional vulnerability, language barriers, and poor role modelling. | Workshops improved students' communication skills; more supervised practice, reflection, and feedback needed to further develop these skills. | [86] |
| **15** | Assessment of empathy among first-year medical students: A knowledge, attitude and practice study. | Quantitative, knowledge, attitude, and practice study. | 100 first-year MBBS students in India. | To assess the empathetic attitude among first-year medical students. | Self-designed, pretested questionnaire administered before summative assessment. | Majority showed low empathy; anatomy dissection contributed to a baseline level of empathy. | Empathy levels were low but improved through anatomy education; strategies are needed to enhance empathy. | [39] |
| **16** | Students’ experiences with a longitudinal skills training program on breaking bad news: A follow-up study. | Longitudinal, mixed-methods (quantitative with student evaluations). | Medical students. | To evaluate how students assess a helical approach to BBN skills training. | Students' opinions were gathered at two time points: immediately after training and after completing clerkships. | Initial satisfaction with training declined after clerkships; students desired more feedback and emotional support. | Early BBN training is valuable, but more feedback and emotional support during clerkships are needed to bridge the gap between training and practice. | [87] |
| **17** | Narrative medicine as a means of training medical students toward residency competencies. | Qualitative, grounded theory. | 12 fourth-year medical students in a narrative medicine elective. | To explore the perceived influence of narrative medicine on clinical skill development (communication, collaboration, professionalism). | Grounded theory approach, using surveys, focus groups, and follow-up interviews. | Students reported improved communication, empathy, collaboration, and professional development; narrative medicine was seen as counter-culture. | Narrative medicine enhances key competencies, but faces resistance; further research needed on its long-term impact and actual clinical performance. | [75] |
| **18** | Clinicians’ strategies for managing their emotions during difficult healthcare conversations. | Qualitative study using self-reported questionnaires. | Clinicians from various disciplines (physicians, nurses, medical interpreters, psychosocial professionals) at a paediatric hospital. | To explore emotion management strategies used by clinicians during difficult healthcare conversations. | Self-report questionnaires prior to simulation-based workshops, open-ended responses analyzed qualitatively. | Clinicians identified five main strategies: Self-Care, Preparatory and Relational Skills, Empathic Presence, Team Approach, and Professional Identity. | Clinicians use varied strategies to manage emotions during difficult conversations; understanding these strategies can inform educational interventions. | [168] |
| **19** | Serious illness communication skills training for emergency physicians and advanced practice providers: a multi-method assessment of the reach and effectiveness of the intervention. | Mixed Methods Study. | Emergency medicine providers across 33 emergency departments. | Assess the reach and effectiveness of EM Talk training. | RE-AIM framework; quantitative analysis of reach, qualitative analysis of post-intervention reflections. | 85% of providers completed the training; improvements in communication skills, attitudes, and practice. | EM Talk training improved serious illness communication and can be integrated into ED curricula. | [169] |
| **20** | Good to great: Using 360-degree feedback to improve physician emotional intelligence. | Conceptual/Review-based (no direct study conducted). | Physicians in healthcare organizations. | Highlight the importance of emotional intelligence (EI) for physicians’ success in healthcare settings. | Literature review and theoretical framework. | EI enhances professionalism, communication, leadership, teamwork, and organizational outcomes in healthcare. | Investing in EI development improves physician effectiveness and alignment in team-based healthcare models. | [110] |
| **21** | Undergraduate students perspective on ethics and communication modules. | Quantitative (survey-based). | 487 undergraduate medical students. | Assess students' perspectives on ethics and communication modules. | Questionnaire distributed via e-learning platform. | 58.5% aware of ethics and communication, 93% support teaching soft skills, 87.3% report improved patient care. | Ethics and communication modules are crucial and should be integrated into medical curricula. | [170] |
| **22** | Medical professional values and education: A survey on Italian students of the medical doctor school in medicine and surgery. | Quantitative (survey-based). | 423 medicine students. | To evaluate students' views on medical professionalism values. | Survey with seven questions; statistical analysis (Chi-square, Wilcoxon, Kruskal-Wallis tests). | High awareness of professionalism values; students want more experience-based learning and integration of values in education. | Medical education should integrate values through experience-based learning, emphasizing empathy, communication, and self-awareness in patient care. | [40] |
| **23** | A failing medical educational model: A self-assessment by physicians at all levels of training of ability and comfort to deliver bad news. | Quantitative, cross-sectional survey. | Pediatric residents, fellows, and attendings at a major academic center. | To explore physician communication skills, particularly in delivering bad news, and identify barriers to improvement. | Voluntary questionnaire assessing self-perceived comfort, knowledge, training, attitudes, and barriers to communication education. | Many trainees and faculty felt unprepared to deliver bad news; barriers included time constraints, lack of emphasis in training, poor role modeling, and limited resource awareness. | Communication skills need more emphasis in training; both didactic and practice-based education are essential for improvement. | [84] |
| **24** | Narrative medicine: Re-engaging and re-energizing ourselves through story. | Qualitative  Study. | Family Medicine residents and interprofessional health care teams. | To evaluate the impact of a Narrative Medicine curriculum on professional development, patient engagement, and wellbeing. | Experiential and didactic workshop, qualitative evaluation of the curriculum's impact. | Narrative Medicine improved residents' professional growth, communication, and patient relationships; increased personal wellbeing. | Narrative Medicine enhances professional development, patient care, and wellbeing; adaptable for other health care settings and teams. | [76] |
| **25** | Qualitative Analysis of a Cultural Dexterity Program for Surgeons: Feasible, Impactful, and Necessary | Qualitative Study. | Surgical residents from three general surgery programs. | To evaluate the feasibility, acceptability, and perceptions of a cultural dexterity curriculum for surgical residents. | Flipped classroom model with e-learning and role-playing, focus groups for feedback, grounded theory analysis. | Five themes emerged: role modeling, relevance, barriers, buy-in, and camaraderie in cultural dexterity training. | The curriculum was feasible, well-received, and highlighted the importance of adaptability; systemic barriers need addressing. | [85] |
| **26** | Deepening the teaching and learning of clinical communication: The importance of reflection and feedback in health education. | Qualitative (conceptual discussion and reflection). | Medical students, health professionals. | Explore reflection and feedback in teaching clinical communication. | Review and conceptual discussion. | Communication requires adaptability; teaching CC needs flexibility and reflection; feedback enhances reflective learning. | Teaching CC should integrate reflection and feedback to develop compassionate, skilled communicators in medical practice. | [81] |
| **27** | Building Telemedicine Capacity for Trainees During the Novel Coronavirus Outbreak: a Case Study and Lessons Learned. | Qualitative case study. | 23 Primary Care Internal Medicine residents. | Evaluate the impact of an adapted telemedicine OSCE on resident training competencies. | Adapted in-person OSCE to a telemedicine format; assessment of competencies in technical proficiency, information gathering, and communication. | Identified training needs in technical proficiency, virtual information gathering, and communication; residents expressed enthusiasm but lacked preparation. | Telemedicine training should address technical, history/exam skills, and communication; further curricular development needed for competencies in virtual environments. | [88] |
| **28** | Implementation of the REFLECT Communication Curriculum for Clinical Oncology Graduate Medical Education. | Qualitative, prospective, educational intervention. | Radiation oncology residents and medical oncology fellows. | To improve and refine physician/patient interactions through a longitudinal communication curriculum. | Implementation of the "REFLECT" curriculum with workshops, role-playing, patient simulations, and reflective writing. | 15 workshops completed; participants had positive perceptions, though faculty felt the impact was greater than trainees. | The REFLECT curriculum enhances communication skills and will be evaluated for its impact on physician interpersonal skills. | [171] |
| **29** | Building Virtual Health Training Tools for Residents: A Design Thinking Approach. | Mixed Methods Study. | 11 residents and 8 attending physicians. | To describe the application of a Design Thinking approach to the identification and co-design of novel training tools to support residents and precepting attending physicians in virtual ambulatory care practice. | The model of “Empathize, Define, Ideate, Prototype, and Test” was applied via a mixed methods approach;  Exploratory interviews and focus groups;  Design Thinking  Workshop (Ideate):  Rapid prototyping (Prototype, Test). | Identified learner needs and problem areas included: improving virtual visit technical skills; acquiring virtual communication skills; adapting to the loss of shared in-person learning space and optimizing virtual learning environments; remediating non-virtual procedural competencies; and educating on new documentation requirements.  Key solution areas included: virtual precepting support tools; digital information and education dissemination tools; and strategies for management of technical issues. Several prototypes were proposed, with a single tool (a virtual preceptor tip sheet) deployed in clinical practice. | Residents found the workshop program improved their understanding of Design Thinking and its relevance to healthcare. | [79] |
| **30** | Developing open disclosure strategies to medical error using simulation in final-year medical students: Linking mindset and experiential learning to lifelong reflective practice. | Qualitative study using focus groups and interpretative phenomenological analysis. | Final-year medical students. | To explore how final-year medical students experience the learning process of open disclosure after a medical error through simulation, linking it to their development of a growth mindset and reflective practice. | The study employed purposive sampling to select final-year medical students for participation in a simulation session on open disclosure. Focus groups were conducted to explore their experiences, and the data were analyzed using interpretative phenomenological analysis (IPA method). | Students constructed their learning through three themes: negotiating environmental relationships, embracing challenge and stress, and achieving learning outcomes. The findings emphasized the importance of psychological safety, emotional arousal, and individual and collective reflective learning. The study also highlighted the connection between experiential learning, growth mindset, and Jarvis's theory. | The study underscored the importance of continuous psychological safety and emotional arousal in learning. It also demonstrated the varied experiences of participants in preparing for open disclosure, emphasizing the need for facilitators to tailor learning to individual and group needs at different stages of learning. | [82] |
| **31** | ‘We work together as a group’: implications of jigsaw cooperative learning. | Explanatory Mixed Method Study. | 1^st^ year medical students at a private university in Malaysia (57 students – survey; 7 students – focus group interview). | To explore the impact of using Jigsaw Cooperative Learning on undergraduate medical students. | Phase I: a survey was conducted to explore the effectiveness of jigsaw learning. Descriptive and inferential statistics were calculated using SPSS.  Phase II: a focus group interview was conducted to explore their in-depth experiences. Qualitative data were thematically analysed. | Quantitative data analysis showed a statistically significant improvement in the student’s individual accountability, promotive interaction, positive interdependence, interpersonal skill, communication skill, teamwork skill, critical thinking and consensus building after jigsaw learning sessions. Qualitative data explained their experiences in-depth. | Jigsaw cooperative learning improves collaboration, communication, cooperation and critical thinking among the undergraduate medical students. | [80] |
| **32** | Human emotion and response in surgery (HEARS): A simulation-based curriculum for communication skills, systems-based practice, and professionalism in surgical residency training. | Comparative Study. | 27 Postgraduate Year 1 (PGY1) residents and 15 Postgraduate Year 2 (PGY2) residents. | To examine the development and implementation of a pilot human factors curriculum during a 2-year period. | Video demonstrations, triggers, and simulated scenarios involving acting patients were created by surgeons and medical educators. Pre and postintervention measures were obtained for communication skills, perceived stress level, and teamwork. Communication skills were evaluated using a series of video vignettes. The validated Perceived Stress Scale and Teamwork and Patient Safety Attitudes survey were used. Residents’ perceptions of the program were also measured. | Significant improvement in empathic communication. There were no significant changes to teamwork attitudes. Perceived levels of stress became considerably higher. | The curriculum is shown to be effective in building communication competency for junior-level residents in the human and emotional aspects of surgical training and practice. | [69] |
| **33** | A comprehensive model for optimizing empathy in person-centered care. | Descriptive Study. | 21 medical professionals representing multiple specialty areas and employed in a teaching hospital, private practice, or clinical setting in Eastern Virginia. | The study examined perspectives regarding the use of empathy within medicine and developed a model to demonstrate the components of empathy in a medical setting. | Grounded theory guided the methodology and final theory formation.  Interviews. | Participant interviews revealed a seven-tier model that displays the facilitative conditions and potential barriers that may impact the full expression of empathy within the medical setting. Interviews also delineated between levels of empathy and described the benefits of providing empathic care, all of which are included in the final model. | This new model of empathy describes a complex and dynamic process and conceptualizes ideal conditions for empathic treatment. | [41] |
| **34** | Perception and reflections of second year medical students on attitude, ethics, and communication skills module on patient autonomy and decision making in a tertiary care teaching hospital. | Prospective, cross-sectional observational questionnaire. | Second-year medical students (n=163) from Vydehi Institute of Medical Sciences and Research Centre who participated in the AETCOM module. | To assess the perceptions and reflections of second-year medical students on the AETCOM module, focusing on patient autonomy and decision-making, and its impact on their understanding of ethical, social, and medico-legal issues in clinical practice. | Two hundred and twenty‑four students were divided into 15 groups with 15 students in each group. There were a totally eight case scenarios, each group was randomly given one case scenario. Students were asked to present their views on the following points sociocultural attitude, ethical issue, medico‑legal issue, and complete the scenario by role‑play.  The study utilized a structured, internally validated questionnaire administered to students after completing the AETCOM module. Reflections were collected from student logbooks. The data were analyzed using descriptive statistics in Microsoft Excel. | The AETCOM module was well-received by students, with an overall mean benefit score of 4.1 out of 5. About 85.3% of students reported improved understanding of sociocultural, ethical, and medico-legal aspects, and 89.6% found panel discussions effective. The mean score for reflections was 6.2 out of 10. | The study concluded that the AETCOM module, which included case scenarios, role-play, and panel discussions, significantly enhanced students' understanding of patient autonomy, ethical dilemmas, and decision-making. It was found to be an effective tool for preparing students for future clinical practice and improving their communication skills. | [93] |
| **35** | A Systematic Review of Educational Interventions and Their Impact on Empathy and Compassion of Undergraduate Medical Students. | Systematic Review. | Medical students. | To conduct a systematic review of research evaluating the associations between spectrum, effectiveness, frequency of teaching modalities and their outcomes on compassion and empathy to highlight best practices. | Data collection from Web of Science, PubMed, Scopus, and EBSCO  2861 initial studies were selected, based on Participants, Intervention (empathy and/or compassion related teaching), Comparison, and Outcome.  After evaluation, 24 articles were analysed. | The authors identified 6 major educational constructs of teaching empathy and compassion; communication, mindfulness, early clinical exposure, technology-enhanced learning, comics and arts and culture. | No standard teaching construct in place was identified and it was highlighted that different teaching tools carry similar impact in promoting compassion and empathy and a sustainable program rather than a single training activity is essential. | [100] |
| **36** | Students meeting with caregivers of cancer patient: Results of an experience-based learning project. | Qualitative research, experience-based learning project. | Third-year medical students from Marmara University and cancer caregivers (relatives of terminally ill cancer patients). | To assess the communication between medical students and cancer caregivers, focusing on the caregivers' experiences with physicians, their expectations for empathic communication, and the impact on students' development of communication skills before starting clinical practice. | The study utilized qualitative methods, including semi-structured interviews conducted by third-year medical students with cancer caregivers. Data were collected through questionnaires and interviews lasting 20–40 minutes. Thematic and content analyses were performed on the collected data. | Caregivers trusted their physicians but expressed a desire for more empathic communication during diagnosis and therapy. Medical students reported development of communication skills, particularly in delivering bad news, through the interviews with caregivers, enhancing their readiness for clinical practice. | The study concluded that the experience-based learning project effectively improved medical students' communication skills, particularly in handling difficult clinical situations and delivering bad news. It also highlighted the importance of empathic communication in enhancing caregiver satisfaction and patient care. | [172] |
| **37** | Effects on Medical Students of Longitudinal Small-Group Learning about Breaking Bad News. | Exploratory Study. | 30 medical students (3^rd^ year) from a Brazilian public university’s medical school. | To examine training in breaking bad news, to improve medical students’ competence and confidence in dealing with this important aspect of clinical practice. | Two focus groups were conducted before the training on breaking bad news, and 2 focus groups were conducted after the intervention was completed, with 15 medical students per group.  In addition, after the intervention, questionnaires were distributed to  the students to assess the importance of training about breaking  bad news and to learn their favorite strategy.  For the content analysis, the Atlas.ti software was used. Descriptive statistics were used to analyse the questionnaires’ respondes.  6-month intervention course about breaking bad news. It included the perspectives of health care professionals, patients, and their families; the SPIKES protocol and the “ABCDE” mnemonic for delivering bad news; general guidelines; and role-playing/simulation strategies to improve students’ skills and reduce their personal limitations. | 30% were aware of the importance of breaking bad news and of the existence of specific protocols to guide physicians in these situations.  90% of students understood the importance and began to apply protocols in their practice. | The knowledge and skills acquired gave the students tools needed to deliver bad news in their future clinical practice. | [173] |
| **38** | Novel approaches to communication skills development: The untapped potential of qualitative research immersion. | Qualitative Study. | In this study, a 17-member multidisciplinary working group of child life specialists, advanced practice providers, undergraduate/medical students, residents, fellows, attending physicians, social scientists, and career researchers with recent qualitative and communication research. | To explore how qualitative research participation impacts clinical trainees’ self-perceived communication skills development and practice. | A modified autoethnographic endeavor in which the collected data from a group of 17 participants  (people with shared qualitative communication research) was analyzed and interpreted collectively.  The sample assembled to discuss this topic using a structured discussion guide. Content analysis was used to identify concepts and themes. | Three key themes characterizing the impact of qualitative research participation on aspiring clinicians’ communication skills development and practice arose – the 3Cs: (1) Connection, therapeutic alliance, and accompaniment; (2) Clarity and prognostic communication; (3) Compassion, empathy, and understanding. Participants emphasized that qualitative research learning improved their understanding of patient/family lived experiences, preparing them for future clinical encounters, strengthening their emotional intelligence, and promoting self-care, resilience, and professional affirmation. | Immersion in clinical communication through participation in qualitative research is an under-utilized resource for supporting clinical trainees in communication skills development. Innovation: The process of collaborative knowledge production through the collective exploration of an a priori question related to group members’ collective experiences is methodologically innovative. | [174] |
| **39** | Telehealth Training for Surgeons to Empathetically Deliver Bad News Via Video-Mediated Communication. | Experience-based learning project. | 33 surgical learners participating in the training. | The goal was to practice new strategies for empathetically delivering difficult information through VMC as a form of telehealth while providing a safe space to practice in a simulated encounter. | Surgical interns (categorical and preliminary), surgical residents, and surgical faculty were recruited to participate in a telehealth simulation-based workshop. Prior to the workshop, all participants were provided information about the case scenario, a video to review best practices for the delivery of bad news using prep-SPIKES, and videos providing examples of both good and poor communication skills.  Each learner was then paired with an SFM in a breakout room with an observing faculty moderator to start a 10-minute VMC encounter where the learner played the role of the physician disclosing the new diagnosis to the SFM, followed by 5-minutes of feedback and reflection. | Only six respondents indicated they had prior formal training on telehealth communication with patients or families, while 13 said they had prior experience giving bad news via telehealth. Comments about improving the training focused on providing more scenarios to practice and more time for feedback. Overall, 25 learners agreed the activity was a valuable learning experience and the majority of responses were positive for future use of telehealth for breaking bad news. | Practicing communication skills with VMC was found to be valuable by surgical interns, residents, and faculty. | [70] |
| **40** | Forum theater staging of difficult encounters with patients to increase empathy in students: Evaluation of efficacy at the University of Angers Medical School. | Quasi-experimental  Study. | 488 fourth-year medical students. | To evaluate whether forum theater (FT) would foster empathy in medical students, and which underlying variables would be associated to empathy scores. | Forum theater was used to explore difficult encounters with patients and family members.  All the students followed two sessions as actors and observers in random order and were randomly assigned to FT sessions after 36 or 56 weeks of clinical rotations.  The Jefferson Scale of Physician Empathy (JSPE) was applied anonymously. | Empathy scores increased after each session.  In regression models, gender and position in the session were significant determinants of JSPE scores, whereas age, session theme, and duration of clinical rotation were not. | Being an actor in forum theater was a valuable tool for enhancing empathy scores in medical students. | [43] |
| **41** | Hidden curricula, ethics, and professionalism: Optimizing clinical learning environments in becoming and being a physician: A position paper of the American college of physicians. | Position Paper. | Physicians. | To provide strategies for revealing what is hidden to foster the development of reflective and resilient lifelong learners who embody professionalism and clinicians who are, and are perceived as, positive role models. | After an environmental assessment to determine the scope of issues and literature reviews, the EPHRC evaluated and discussed several drafts of the paper. The paper was then reviewed by members of the ACP Board of Governors, Board of Regents, Council of Resident/Fellow Members, Council of Student Members, and other committees and experts. | The best solutions to the influence of the hidden curriculum will uncover it, integrate its positive aspects into the formal curriculum, and lead to development of approaches to understand and mitigate its negative aspects by educators and practicing clinicians. The hidden curriculum presents challenges but also opportunities to help reshape the education and culture of medicine. | The educational and social milieu of medical learning environments is a complex system of influences that include role models across peer relationships and the hierarchy of medicine contribute to the formation of professional identity, behaviors, and attitudes of future physicians. | [118] |
| **42** | Doing the hard work of learning: oncologists’ enduring impressions of a year-long communication skills training program. | Cross-sectional survey. | 114 medical oncology fellows. | examined the long-term impact of communication skills training for oncologists. | Curriculum  Online survey. | Forty-one (60%, 95% CI: 49.3–73.8) fellows strongly  agreed or agreed with item 12, “Difficult Conversations had  a profound impact on the way I practice medicine.” Fortythree  (64%, 95% CI: 51.5–75.5) fellows strongly agreed or agreed with question 14, “I often find myself informally  sharing lessons I learned during the series.” Fifty-seven  (84%) fellows agreed or strongly agreed that “Psychosocial  education and communication skills training for medical  oncology fellows is underemphasized and should receive  more attention.” Fifty-one of the 55 fellows (93%, 95% CI:  69.1–89.8) who entered training in 2012 or later found the  video session with the standardized patient at the beginning  of the year to be useful and relevant. | most oncologists who participated  in “Difficult Conversations” perceived that the  curriculum helped them acquire important skills, improved  their confidence with specific challenges, and profoundly  impacted their medical practice. | [66] |
| **43** | Patient-oriented learning: A review of the role of the patient in the education of medical students. | Literature review. | Medical students. | To explore the contribution patients can make to medical education from both theoretical and empirical perspectives, to describe a framework for reviewing and monitoring patient involvement in specific educational situations and to generate suggestions for further research. | Literature review. | Direct contact with patients can be seen to play a crucial role in the development of clinical reasoning, communication skills, professional attitudes and empathy. It also motivates through promoting relevance and providing context. Few studies have explored this area, including effects on the patients themselves, although there are examples of good practice in promoting more active participation. | The Cambridge framework is a tool for evaluating the involvement of patients in the educational process, which could be used by curriculum planners and teachers to review and monitor the extent to which patients are actively involved. | [175] |
| **44** | Assessing students' communication and interpersonal skills across evaluation settings. | Observational Study. | 127 medical students. | To observe if medical students' communication skills improve between their third-year family medicine exam and fourth-year clinical practice exam by comparing scores from standardized patients. | 127 students completed both the clerkship OSCE and a CPX. The CPX was a pilot of the National Board of Medical Examiners Standardized Patient Exam. To assess students’ interpersonal skills, both exams used modified versions of the American Board of Internal Medicine Patient Satisfaction Questionnaire. Students’ scores were standardized, and correlations were calculated. A logistic regression model examined the ability of the OSCE to predict poor performance on the CPX. | The correlation between the OSCE and CPX patient satisfaction scores was *.08*. There was no significant predictive ability of the OSCE for poor performance on the CPX. | The study calls into question the ability of a routine end-of-clerkship OSCE to identify students’ interpersonal skills abilities on fourth-year clinical performance exams and potentially that component of the anticipated USMLE SP exam. | [157] |
| **45** | Assessing competence in communication and interpersonal skills: The Kalamazoo II report. | Comparative Review Study. | Educators, evaluators, and researchers. | To summarize the methods and tools used by educators, evaluators, and researchers in the field of physician–patient communication as determined by the participants in the  “Kalamazoo II” (April, 2002). | Reporting and summarizing the methods and tools mentioned in the Kalamazoo  II conference. | 1) The same assessment tool may be used for formative evaluation and feedback during training or for summative and high-stakes evaluations for promotion, licensing, or certification;  2) Demonstration of interactive skills demands observation and ratings of real or simulated physician–patient encounters;  3) Selection of the tools chosen by an educational or evaluation program will depend on the resources available and the level of reliability and validity required;  4) the therapeutic essence of the doctor–patient relationship should include the patient’s perspective obtained either from ratings or surveys after encounters. | The Kalamazoo II report elaborates on competence in interpersonal skills, concluding with a summary of the assessment tools that may be used for teaching and evaluating these competencies. | [92] |
| **46** | A national survey to define a new core curriculum to prepare physicians for managed care practice. | Descriptive Study Survey. | Different groups of medical school directors and representatives; care practitioners, administrators, and educators. | To sought information from a variety of sources to serve as a basis for identifying the core curricular components and the staging of these components in the medical education process. | Appliance of three surveys: one for  125 U.S. medical school curriculum deans or equivalent representatives; other to four focus groups of managed care practitioners, administrators, educators and residents; and a third survey of a national sample of physicians and medical directors. | Almost all the 91 responding school representatives recognized the importance of revisiting their curricula to meet the managed care challenge and that the majority either had or were developing programs to train students for practice in managed care environments. The focus groups identified a core set of competencies for managed care practice, although members differed on whether the classroom or a managed care setting was the best place to teach the components of a new curriculum. Although medical directors and staff physicians differed with respect to the relative levels of importance of these competencies, the findings suggest that before medical school, training should focus on communication and interpersonal skills, information systems, and customer relations; during medical school, in clinical epidemiology, quality assurance, risk management, and decision analysis; during residency, on utilization management, managed care essentials, and multidisciplinary team building; and after residency, on a review of a customer relations, communications skills, and utilization management. | A core curriculum and its sequencing can be identified that the majority of curricular components exist but, in some cases, need to be modified more clearly relate to managed care practice, and that their findings may provide a useful starting point for making decisions about curricular reform. | [146] |
| **47** | Difficult conversations in health care: Cultivating relational learning to address the hidden curriculum. | Descriptive Study. | Health care professionals. | The Program to Enhance Relational and Communication Skills (PERCS) aims to embody and promote new and expanded definitions of professional competence in the medical world | Description of the program’s rationale, goals, value premises, structure, and format. Exploration of how the program works, by  providing an in-depth account from a typical workshop. Reflection on why the program works, by exploring key features of the author’s pedagogical approach. | The program responds to several  developments in contemporary health  care: medical education reform,  changing definitions of professional  competence, and calls for greater  attention to qualities of compassion,  trust, and respect in practitioners’  relationships with patients and families. | It is possible, in the busiest  of hospital environments, to fashion learning experiences that promote moral reflection and reconnection with one’s  humanness, and that participation in  this kind of relational learning can be transformative, both personally and professionally. | [152] |
| **48** | Educating for empathy: A review. | Literature review. | Undergraduate medical students. | To identify effective strategies to enhance empathy in undergraduate medical students. | Literature aligned with the topic was retrieved from PubMed: 13 peer-reviewed, English language, qualitative and quantitative studies were identified, reporting primary data on interventions that aim to foster empathy, using Medical Subject Heading terms education, medical, undergraduate or student, medical crossed with empathy. | Empathy may be amenable to positive change with a range of interventional strategies. Communication skill workshops addressing the behavioral dimension of empathy show greatest quantitative impact on participants. However, current studies are challenged by varying definitions of empathy, small sample sizes, lack of adequate control groups, and variation among existing empathy measurement instruments. | Given the methodological limitations of the available studies, and uncertainty about which dimensions of empathy should be addressed, larger studies using validated measurement tools are recommended. | [44] |
| **49** | Expanding clinical empathy: An activist perspective. | Literature Review. | Physicians. | To discussion empathy in health care, taking into account the component for alleviating the patient’s suffering and emphasize the physician’s experience of empathy rather than the patient’s experience of illness. | Examination of educational methods (reflective writing exercises); study of literary texts; analysis of theoretical approaches to empathy and suggestions for clinical practice. | Clinical empathy can be improved by strategies that address (1) the patient’s authority in providing first-person accounts of illness and disability, (2) expanding the concept of empathy to include an action component geared toward relieving patients’ suffering, and (3) the value of extending empathy to include the social context of illness. | Future discussions of and exercises in empathy will benefit from emphasizing patients’ points of view and the uniqueness of patients’ experiences, as well as the community and social context that encompasses physicians and patients. | [45] |
| **50** | Impact of clinical interview training on the empathy level of medical students and medical residents. | Comparative Evaluation Study.  Quasi-experimental pre-test/post-test study of 203 subjects. | 137 second-year medical students at the University of  Castilla-La Mancha and 66 medical residents at the Family  and Community Medicine Teaching Unit of Albacete, Spain. | Assess the impact of a communication skills workshop on the empathy level of medical students and medical residents. | Jefferson Scale of Physician Empathy (JSPE)  2 groups: control group (20 residents, 55 students) and experimental group (46 residents, 82 students) that participated in a 25-hour workshop on communication and empathy. | The mean pre-workshop JSPE score was similar in both groups. No significant increase in JSPE score after the second assessment was observed in the controls.  Results showed a similar empathy level among students  and residents, but with gender-related  differences (women students had higher scores than men). | A communication skills workshop yields a slight improvement of crucial practice importance in subjects’ empathy.  Professionals should be receptive to opportunities offered  by patients to show empathy. | [46] |
| **51** | Effects of a course on ophthalmologist communication skills: A pilot study. | Pilot Study. | 11 ophthalmologists. | The study aimed to discuss the effects of a 16-hour communication skills course for ophthalmologists and to assess the interest of participants with respect to the topic and the efficacy on participant’s communication skills, at least in a laboratory setting. | Learner satisfaction: questionnaire with a six-point Likert scale.  Course efficacy: comparison between communicative behaviour of ophthalmologists in videoed role playing before and immediately after attending the course. Videoed consultations were coded using the Patient Centred Score Sheet (PCSS) and the Roter Interaction Analysis System (RIAS). The Wilcoxon signed rank test was used for statistical analysis. | The course obtained high satisfaction in participants (mean score *5.1*). In the post-test role playing, patient centredness increased significantly (p < *0.01*). Furthermore, ophthalmologists improved their competence in using open ended questions (p < 0.02), process categories (e.g. orientation statement) (p < *0.05*) and social communication categories (e.g. personal statement) (p < *0.01*). | Ophthalmologists showed satisfaction for the course, which positively influence ophthalmologist communication competence, at least in a laboratory setting. | [71] |
| **52** | Prior experiences associated with residents' scores on a communication and interpersonal skill OSCE. | Exploratory, cross-sectional, observational study. | 79 second- and third-year medical residents (PGY 2-3) from Internal Medicine and Family Medicine programs. | To explore whether prior task experience and comfort levels correlate with performance on an Objective Structured Clinical Examination (OSCE) assessing patient-centered communication skills in medical residents. | A six-station OSCE was used to assess communication skills, and a survey collected information on residents’ prior experiences. Statistical analyses (t-tests, correlations, multi-factorial ANOVA) were applied to explore relationships between prior experience and OSCE scores.  A survey provided information on prior experiences. t-tests, correlations, and multi-factorial ANOVA explored relationship  between scores and experiences. | 1. Prior experience with tasks influenced comfort but did not improve communication scores. 2. Comfort was moderately correlated with scores in some cases, but increased comfort did not guarantee better performance.  3. Female residents and those with standardized patient experience in medical school had higher scores.  4. Residents without medical school standardized patient experience were almost five times more likely to be rejected by patients in the OSCE. | Prior task experience does not guarantee improved communication performance, as it may lead to overconfidence. However, standardized patient experiences during medical school and patient-centered interviewing courses were associated with better performance. These experiences can contribute to long-term improvements in communication skills. Further research is needed to evaluate the effects of these experiences.  The combination of didactic  courses and practice with standardized patients may be an  effective way to encourage a patient-centered approach. | [67] |
| **53** | Emotional intelligence and clinical skills: Preliminary results from a comprehensive clinical performance examination. | Exploratory, correlational study. | 165 third-year medical students (from 2003 and 2004 cohorts). | To examine the relationship between emotional intelligence (EI) and clinical skills, specifically communication and physical examination skills, as assessed by standardized patients during a comprehensive performance examination (CPX). | Students completed the Trait Meta-Mood Scale (TMMS) and Davis' Interpersonal Reactivity Index (IRI) prior to taking a 12-station CPX. The subscales of these instruments were correlated with students' clinical skills as recorded by standardized patients. | 1. Attention to Feelings (TMMS), Empathic Concern, and Perspective Taking (IRI) were positively correlated with communication skills.  2. Empathic Concern and Perspective Taking were negatively correlated with physical examination skills.  3. The internal consistencies of the subtests ranged from 0.73 to 0.90. | The study suggests that various aspects of emotional intelligence, particularly empathy-related dimensions, are modestly related to students' clinical skills in an OSCE. While some empathy subscales were associated with communication skills, they showed negative correlations with physical examination performance, suggesting the need for further exploration of these relationships. | [147] |
| **54** | Development of a Communication Curriculum for Emergency Medicine Residents. | Observational and Retrospective Study. | Twenty-six residents in  a 3-year program Emergency medicine (EM) residents. | The authors piloted a communication curriculum for emergency medicine residents. In the paper they describe the curriculum and suggest future directions for development based on the strengths and weaknesses of residents’ performance and their reactions to the curriculum. | Twenty-six residents in a 3-year program at a university-affiliated county hospital participated. Curriculum components were an introductory session, a single standardized patient encounter using a locally written, unvalidated checklist assessing residents’ communication skills, a videotape-facilitated self-assessment, and a private feedback session. | Residents demonstrated greatest strengths in basic interpersonal skills and efficient information gathering and greatest weakness in empathy. Residents rated the curriculum favourably. | The curriculum as implemented offers an initial foundation for teaching and learning critical care communication. Instruction in empathy requires improvement. | [47] |
| **55** | Teaching communication skills in clinical settings: comparing two applications of a comprehensive program with standardized and real patients. | Comparative Study. | Healthcare professionals (25 in the first year, 20 in the second year). | The study inspected whether (1) results from a communication program are replicated with different samples, and (2) results with standardized patients apply to interviews with real patients. | A structured, nine-month communication program was applied in two consecutive years to two different samples. Results were assessed at four different points in time, each year, regarding participants’ confidence levels (self-rated), basic communication skills in interviews with standardized patients, and basic communication skills in interviews with real patients. Data were analyzed using GLM Repeated-Measures procedures.  Observational study, videotaped interviews. | Improvements were statistically significant in both years in all measures except in simulated patients’ assessment of the 2008 group. Differences between the two samples were non-significant. Differences between interviews with standardized and with real patients were also non-significant. | The program’s positive outcomes were replicated in different samples, and acquired skills were successfully applied to real-patient interviews. | [72] |
| **56** | Assessing empathy development in medical education: a systematic review. | Systematic Review. | Examined physicians,  medical students or residents. | To learn how empathy is conceptualised in medical education research. To examine how researchers define the central construct of empathy and what they choose to measure, and investigated how well definitions and operationalisations match. | Systematic review PubMed; EMBASE; Scopus; Cumu-  lative Index to Nursing and Allied Health  (CINAHL); Web of Science; PsycINFO, and the Edu-  cation Resource Information Center (ERIC). | 109 studies that met search criteria, however 20% failed to define the central construct of empathy at all and only 13% used an operationalisation that was well matched to the definition provided. Most studies were characterised by internal inconsistencies and vagueness in both the conceptualisation and operationalisation of empathy, constraining the validity and usefulness of the research. | The authors suggest that future research should follow the lead of basic scientific research that conceptualises empathy as relational – an engagement between a subject and an object – rather than as a personal quality that may be modified wholesale through appropriate training. | [48] |
| **57** | Empirical research on empathy in medicine-A critical review. | Critical/ Systematic Review. | Medical students and physicians. | empirical research on empathy in medicine -  To assess methodological limitations in this body of research that have not received adequate attention. | A systematic literature search in Ovid MEDLINE(R), PsycINFO,  EMBASE, and CINAHL was performed.  Scientific publications presenting empirical research on medical students’ or physicians’ empathy were systematically searched ~~for.~~ | 206 publications were critically reviewed. Multiple empirical approaches have been used. However, there are some remarkable tendencies given the complexity of the study object: empathy is often not defined. Qualitative approaches are rarely used and the predominant quantitative instruments have a relatively narrow or peripheral scope. For example, the concrete experiences, feelings, and interpretations of the physician and the patient, and empathy in clinical practice, are often neglected. Furthermore, possible influences of medical training and working conditions on empathy have not been adequately explored. | The empirical studies of empathy in medicine tend to separate empathy from main parts of clinical perception, judgment, and communication. Thus, important aspects and influences of empathy have been relatively neglected. | [49] |
| **58** | Measuring Medical-Students Empathy Skills. | Randomized Controlled Trial. | Sixty first-year clinical medical students from the Austin and Repatriation Hospitals’ Clinical School. | To review approaches to measuring empathy in medical education and evaluate the effectiveness of a two-stage measurement technique, including a pencil-and-paper test of empathy and independent observer ratings of students’ interview behaviors during videotaped history-taking sessions with hospitalized patients. | A randomized controlled study design was employed. Students were divided into experimental and control groups. The experimental group received training in consulting skills aimed at enhancing empathic abilities. Empathy was assessed using a pencil-and-paper test and trained observer ratings based on a History-taking Rating Scale during videotaped interviews. | Pencil-and-paper tests of empathy were found to be insufficient for capturing the full complexity of empathy, which includes cognitive, emotional, and behavioral components. Trained observers using the History-taking Rating Scale effectively discriminated between empathic behaviors of the experimental group (trained in consulting skills) and the control group. | Consulting skills training enhances medical students’ empathic abilities as demonstrated in observed behaviors during patient interviews. Pencil-and-paper tests alone are inadequate for assessing empathy due to its multifaceted nature, whereas observer-based ratings provide a more comprehensive assessment. | [50] |
| **59** | Teaching empathy in medical education. | Quasi-experimental  Study.  Pre-Test and Post-Test Design: Empathic Tendency Scale (ETS) and Instrument for Attitudes Towards Communication in Medicine (IATCM). | First-year students of Adnan Menderes University Medical School in the academic year 2003-2004. Participants included 62 students in the pre-education period and 36 students in the post-education period. | To assess differences in empathic tendencies and attitudes toward communication in medical practice among first-year medical students and to evaluate the impact of education on these factors. | Empathic Tendency Scale and Attitudes Towards Communication in Medical Practice Questionnaire.  Students were clustered into two groups for both scales using K-means analysis, which was used to see whether the students were homogeneous regarding empathy and communication attitudes at the beginning of the teaching period. | Students are clustered into two groups for both scales using K-means analysis, which was used to see whether the students were homogeneous regarding empathy and communication attitudes at the beginning of the teaching period. According to the Empathic Tendency Scale results 25% of students have a high empathic tendency (HET) and 75% have a low empathic tendency (LET) and the results of the Attitudes Towards Communication in Medical Practice Questionnaire indicated that 56% have positive and 44% have negative attitudes towards communication. However, the difference between the pre-and post-teaching results of either group seems to show a tendency towards change (p< 0.10). The change has a negative and a positive tendency in the HET and LET groups, respectively. | The study confirmed differences among students in empathic tendencies and communication attitudes. Empathic tendency appears more modifiable through targeted teaching techniques, especially when considering changes within groups. | [51] |
| **60** | Empathy variation of undergraduate medical students after early clinical contact: a cross-sectional study in China. | Quasi-experimental Study.  Pre-Test and Post-Test Design: Jefferson Scale of Empathy -Student version (JSE-S). | A total of 257 undergraduate medical students participated in the study. The 154 first year students were studying in 10 community-based teaching hospitals, and the 103 second-year students were studying  in 3 university affiliated hospitals. | To compare the self-reported empathy levels of undergraduate medical students before and after their early clinical contact curriculum. | Primary measures: the Jefferson Scale of Empathy-Student  version (JSE-S)  was compared between students of different sexes and in different academic years before  their early clinical contact course. Secondary measures:  comparisons were made after they finished the curriculum  3 weeks later. | A total of 219 of 257 students responded  (85.21% response rate), and 214 answers could be  analysed (135 first-year  and 79 second-year students; 120  female and 94 male individuals). No significant differences  in the empathy scores before early clinical contact were  observed between students of different sexes and in  different academic years. After early clinical contact, the mean JSE-S  score of the participants was significantly higher than the mean score at the beginning of the  curriculum. | Empathy-focused  training during early  clinical contact can improve the empathetic capacity of  undergraduate medical students. Fostering empathetic  attitudes among undergraduate medical students is  necessary for the early stage of their medical education.  Further research is needed on the long-term effects  of empathy-focused  education in entry level medical students. | [52] |
| **61** | Teaching Shared Decision Making to Undergraduate Medical Students. | Educational Intervention | Undergraduate medical students. | To promote the integration of shared decision-making (SDM) into clinical practice by teaching it to undergraduate medical students, with a focus on developing empathy and understanding patient concerns, rather than just conveying health-related information. | Proposed curricular design with smaller, sequential units focusing on patient concerns and decision-making involvement.  Emphasis on empathy, communication skills, and active listening.  Scenario-based learning and patient-centered approach. | The proposed approach is expected to improve medical students’ ability to understand and address patients' concerns and preferences in decision-making.  It emphasizes empathy and patient-centered communication  Expected benefits include fostering patient-centered decision-making, improving communication skills, and enhancing doctor-patient relationships. | Teaching SDM to undergraduate students, focusing on empathy and understanding patient concerns, will lead to better doctor-patient relationships and more effective shared decision-making in clinical practice. | [53] |
| **62** | A Multifaceted Educational Intervention in the Doctor-Patient Relationship for Medical Students to Incorporate Patient Agendas in Simulated Encounters. | Observational Study. | 109 medical students prior to their clerkships. | To assess whether medical students increased their sensitivity towards patients' needs and improved their ability to conduct bidirectional conversations after an instructional programme. | Observational study; five-step training programme with experiential and reflective strategies to encourage communication skills (CSs). | Improvement in communication skills across three sessions as rated by both external observers (EO) and simulated patients. 83.9% of students found CSs useful for clinical practice. | The programme helped students use communication skills for bidirectional conversations in simulated settings. It is feasible to integrate these skills into broader training. Further research is needed for real-world applicability and other outcomes. | [56] |
| **63** | Mindset and Reflection-How to Sustainably Improve Intra- and Interpersonal Competences in Medical Education. | Qualitative Study. | 21 medical experts. | To detect factors for the sustainable implementation of intra- and interpersonal competences (IICs) in medical education. | Interviews analyzed according to grounded theory principles to generate core categories addressing the research questions. | IICs are changeable and developable in many students. Four central prerequisites for successful implementation were identified: integration of reflection and feedback, clear framework and individual path, students’ mindset for personal development, and superiors’ mindset for addressing personal challenges. | The development of a mindset that allows open discussion of personal deficits and challenges is central for both students and teachers. Educating teachers and superiors could help improve IICs in medical professionals. | [176] |
| **64** | How does narrative medicine impact medical trainees' learning of professionalism? A qualitative study. | Qualitative Study. | Thirty medical trainees, including five fifth-year medical students (MSs), ten sixth-year MSs, nine seventh-year MSs, and six postgraduate year (PGY) trainees. | To explore how narrative medicine (NM) impacts medical trainees' learning of professionalism during a clerkship in a Taiwanese clinical setting. | A qualitative interview study using purposive sampling. Thematic framework analysis and a modified realist evaluation approach were employed to analyze the data. | Self-exploration, reflection, and awareness of professional identity were mechanisms explaining how NM impacted professionalism learning. Outcomes included empathy, communication, doctor-patient relationship, and understanding patients. | NM facilitates self-exploration, reflection, and awareness of professional identity, enhancing trainees' professionalism learning. NM can play an important role in undergraduate medical education. | [77] |
| **65** | Interpersonal behaviors and socioemotional interaction of medical students in a virtual clinical encounter. | Exploratory Observational Study. | Thirty medical students (sixth semester), participating voluntarily in the study. | To investigate the dynamics and congruence of interpersonal behaviors and socioemotional interaction during a virtual clinical encounter (VCE), and to evaluate which interaction design characteristics contribute most to behavioral and affective engagement in medical students. | Exploratory observational study with a highly interactive virtual patient (VP) case. Medical students worked in pairs, were videotaped for behavioral analysis, and completed surveys and interviews for self-reporting opinions and perceptions. A mixed methods approach was used. | Participants demonstrated adequate clinical case management and obtained psychosocial history. Collaborative work fostered dynamic discussions. The VCE was perceived as a motivational and engaging learning environment, enhancing interpersonal communication skills. | VCEs promote not only critical reflection and strategy-selection skills but also improve listening, nonverbal communication, self-awareness, and coping behaviors. Early application of VCE in medical education may enhance clinical encounters and responsible decision-making. | [73] |
| **66** | “An invitation to think differently”: a narrative medicine intervention using books and films to stimulate medical students’ reflection and patient-centeredness. | Mixed Methods Study. | Fourth-year medical students at a Dutch medical school. | To evaluate the impact of a mandatory narrative medicine lesson on stimulating meaningful self-reflection on patient-centeredness among medical students. | Mixed-methods design: qualitative analysis of 203 reflective essays and quantitative analysis using the REFLECT rubric. Students participated in a mandatory narrative medicine lesson, consisting of an introductory lecture, reading and watching materials, discussion groups, and reflective writing exercises. | Students demonstrated reflection on both professional and personal levels. About half of the students showed in-depth and authentic reflection. Reflection levels varied based on thematic pairings (book and film). | Narrative medicine enhances self-reflection and self-awareness among students, contributing to patient-centered care. The exercise supports the development of attitudes toward patient-centeredness and understanding patient perspectives. | [78] |
| **67** | Using patient storytelling to improve medical students’ empathy in Japan: a pre-post study | Quasi-experimental Study.  Single-group pretest-posttest comparative study. | 159 fourth-year undergraduate medical students in Tokyo, Japan (academic years 2018 and 2019). | To quantitatively evaluate how patient storytelling enhances empathy in medical students and whether this empathy is sustained for six months. | Participants completed the Jefferson Scale of Empathy-Student Version (JSE-S) questionnaire at three time points: before, immediately after, and six months after the class. Gender, age, and clinical orientation data were also collected. A male patient with chronic kidney disease participated in storytelling during “Professionalism” classes. | JSE-S scores improved significantly immediately after the storytelling session, with improvements sustained six months later. Interest in specialty was positively associated with immediate score changes. Gender did not affect empathy score changes. | Patient storytelling is effective in cultivating empathy in medical students. It is anticipated that more medical schools will adopt this method for humanistic and communication education. | [54] |
| **68** | Narrative medicine in clinical internship teaching practice. | Randomized Controlled Trial (RCT). | Five-year clinical medicine senior undergraduate medical students engaged in practice in the Department of Obstetrics and Gynecology at the Third Xiangya Hospital of Central South University in 2019.  A total of 100 students, randomly divided into two groups: control group (n = 50, 24 females) and experimental group (n = 50, 28 females). All students were taught by the same teacher. Participation was voluntary, and students were provided with an information sheet about the study and opt-out options. | To explore the effect on empathy skills of integrating narrative medicine instruction  into clinical internship undergraduate medical education. | One hundred clinical undergraduate students who were transferred to gynecology  and obstetrics in 2016 were selected as subjects and divided into two groups. The control  group adopted the traditional practice teaching mode, while the experimental group  adopted a narrative medicine integrated with traditional teaching mode. The impact of the  narrative medicine course was evaluated using the Davis Empathy Scale, and the students’  acceptance of the course was investigated using a self-developed questionnaire. | After completion of the rotation, the empathy scores of the experimental group  were higher than those of the control group (p < 0.05). Students in the experimental group  rated the integration of narrative medicine into the internship class highly, and most students  thought that the narrative medicine course was of great benefit with respect to the humanistic  quality of medical teaching. | The application of narrative medicine teaching in the clinical practice teaching of  obstetrics and gynecology promoted students to improve their empathy ability. | [55] |
| **69** | Virtue and care ethics & humanism in medical education: a scoping review. | Literature Review. | Medical students, healthcare professionals. | To explore how virtue and care ethics are incorporated into health professions education and their relationship to humanistic patient care. | Scoping review methodology involving identification of relevant citations across several databases (PubMed, NLM Catalog, WorldCat, etc.), applying inclusion and exclusion criteria, and systematically analyzing articles based on defined steps. | 811 citations identified, 88 intensively reviewed, and 25 analyzed in-depth. Multiple key themes and metaphors related to virtue/care ethics, curricula, and humanism in education were found. | Virtue and care ethics have potential to promote humanism in healthcare, with themes that facilitate or impede the integration of these concepts into curricula. | [57] |
| **70** | The Impact of an Unconventional Elective in Narrative Medicine and Pediatric Psycho-oncology on Humanism in Medical Students. | Qualitative study. | Third- and fourth-year medical students participating in an immersive elective in pediatric psycho-oncology. | To evaluate the impact of an immersive elective in pediatric psycho-oncology coupled with narrative medicine on medical students. | Qualitative evaluation through a retrospective analysis of 120 narrative journals written by students between 2008 and 2019. Participants were relieved of traditional clinical duties and shadowed pediatric cancer patients, reflecting on their experiences in journals. | Students developed a deeper understanding of patient experiences, a humanistic view of illness, and meaningful reflections on interactions with critically ill children. Families appreciated the students' emotional attentiveness. | Experiential learning combined with narrative reflection helps students regain empathy and provides valuable insights, potentially enhancing humanism in medical education. Further longitudinal and quantitative studies are needed to assess the long-term benefits. | [63] |
| **71** | The physician healer track: educating the hearts and the minds of future physicians. | Mixed Methods Study. | Medical students, specifically those participating in The Physician Healer Track (PHT), with an enrollment growing from 26 students in the first year to 258 students across six years (20–26% of each class). | To provide a comprehensive, voluntary program aimed at developing humanistic, compassionate physicians through training in professional identity formation, self-awareness, communication, mindfulness, and reflective practices. | The voluntary scholarly concentration program comprises 4 years of monthly dinner meetings with faculty mentors, a two-month preceptorship in the first year, a one-month immersion course in MS4 and one elective. Training in mindfulness, cognitive behavioural therapy, nonviolent communication, motivational interviewing, spirituality in healthcare, wellness, equanimity, and ‘being with suffering’ is reinforced across all 4 years. Community building and reflection are integral to the training both in the monthly sessions and the immersion courses. | Graduates in the first two cohorts of PHT have exceeded the numbers in the eight other scholarly concentrations offered at UTMB. Among students participating in the summer preceptorship, there has been less than 1% attrition. In serial assessments, students report continued growth in personal development, professional development, and the ability to empathize. Offering PHT has resulted in the growth of training for our medical residents, faculty, physical therapy students and the creation of a student healer association. | The program contributes to students' growth, fosters community-building, and enhances the training of medical residents and faculty. Students voluntarily engage in the program despite its demands, aiming to maintain their humanity throughout training. | [177] |
| **72** | Humanism in clinical education: A mixed methods study on the experiences of clinical instructors in Iran. | Qualitative Study. | Medical Students. | The present qualitative  study aimed to explain the experiences of clinical professors with regard to humanism in clinical education in Iran. | This mixed methods study had two phases, a quanitative phase of scientometrics and a qualitative phase  of a content analysis. In the scientometrics phase, Ravar PreMap and VOSviewer software programs were utilized for  plotting the conceptual networks. The networks were analyzed at the micro-level based on centrality indices  (closeness, degree, and betweenness). The conceptual network was plotted and the prominent topics in clinical  education were identified using co-word analysis. In the second qualitative phase on the topic, based on the  scientometrics phase, semi-structured interviews were conducted with clinical professors. The interviews were  transcribed verbatim and analyzed. | On the basis of the analysis of titles, abstracts, and keywords of the retrieved articles on clinical education  from ISI Web of Science, Scopus, and PubMed, 1412 keywords were extracted. After the refining process, 356  keywords with 6741 relations remained. Upon plotting the conceptual network, 19 conceptual clusters related to  clinical education were obtained. Then, micro-level network analysis (centrality criteria) indicated that the keyword  humanism with the frequency of 137 had the highest rate (97.753), closeness (97.802), and betweenness (13.407).  Moreover, from the interview data analysis, two themes of “intertwined nature of the human spirit in clinical  education” and “humanistic behavior of professors in clinical education” were extracted. | As a part of the educational culture, humanistic values must be intertwined with the medical  education curriculum. In this regard, humanism and clinical reasoning are the two major clusters of clinical  teaching; moreover, altruism and adherence to humanistic values, and scientific qualification are other main pillars  that should be considered as the criteria for the selection of clinical professors and medical students. | [58] |
| **73** | Unveiling the hurdles in cultivating humanistic physicians in the clinical setting: An exploratory study. | Qualitative study. | Medical students in the clinical phase, residents, clinical teachers, and module administrators at Universitas Indonesia's Academic Health System (AHSUI). | The study aims to explore the challenges in cultivating humanistic physicians in the clinical setting, with a focus on the role of medical education in shaping the humanistic values of physicians. | A qualitative study using a phenomenological design. Data were collected through eight focus group discussions (FGDs) and two in-depth interviews, involving clinical-phase medical students, residents, junior and senior clinical teachers. Thematic analysis was used to analyze the data. | Respondents from different groups of stakeholders shared the same definition  for ‘humanistic physician’: a physician who provides patient-centred care while demonstrating  empathy, respect, compassion, integrity, knowledge, competence and a collaborative spirit.  Despite changes in the healthcare system and technological advancements, humanistic physicians  are still needed. | Cultivating humanistic physicians is a complex process, requiring various  methods and assessments. Role models play a significant role in this process, which included not  only clinical teachers but also peers. Feedback from peers was perceived as an important factor.  The key hurdles identified were negative role models, and a less humanistic learning environment  and the students’ personal backgrounds. | [178] |
| **74** | Humanistic medicine in anesthesiology: development and assessment of a curriculum in humanism for postgraduate anesthesiology trainees. | Quasi-Experimental Study. | 10 clinical anaesthesia Year 1 residents (CA-1) and 60 patients (30 before implementation and 30 after implementation) undergoing elective surgery. | To develop, implement, and evaluate a humanism curriculum in perioperative medicine, focusing on improving empathy and professionalism in anaesthesiology training. | Subject-matter experts developed and delivered the humanism curriculum, which included interactive workshops, simulation sessions, formal feedback, and patient immersion experience. The effectiveness of the programme was evaluated using pre- and post-curriculum assessments. | The anaesthesiology residents reported high satisfaction scores. Pre-/post-Jefferson Scale of Patient Perceptions of Physician Empathy showed an increase in empathy ratings. After training, patients rated the residents as more empathetic and professional. Patient overall satisfaction with their anaesthesia provider improved after training. Patients rated their anxiety lower in the post-training period compared with pretraining. Patient-reported pain scores decreased after training. | The humanism curriculum in perioperative medicine was well-received and led to enhanced resident empathy and professionalism, contributing to improved patient satisfaction and care outcomes. | [59] |
| **75** | Linking the heart and the head: Humanism and professionalism in medical education and practice. | Qualitative (conceptual discussion and reflection). | Health professionals. | To develop a model that reflects the complex processes and catalysts for humanistic practice and education, while being straightforward to be recalled and used in real life. | A review of articles from multiple databases (MEDLINE, ATLA, PsycINFO, ERIC, Google Scholar) on humanism in medical education, followed by thematic analysis and iterative development of a conceptual model | A conceptual model was developed, which centers on the interaction between cognitive domains (head) and emotive domains (heart) of humanism. Four provinces of experience (personal reflection, action, system support, and collective reflection) nurture humanism. | Humanistic development in medical education and practice can be fostered through critical reflection, conscious awareness, and supportive systems that connect the heart (emotive domains) and head (cognitive domains). | [60] |
| **76** | A study of core humanistic competency for developing humanism education for medical students. | Observational Studies. | The participants included 1,132 individuals: 154 medical school professors (including adjunct professors), 589 medical students (including pre-medical students), 228 parents, and 161 college and hospital staff (such as nurses and laboratory technicians) from Seoul National University College of Medicine, Korea. | To assess the essential humanistic competencies that medical students should possess and identify the most effective teaching methods to develop these attributes. | A survey with nine questions was conducted across multiple groups: medical professors, students, parents, and staff. Participants rated humanistic competencies (morality, ethics, empathy, communication skills, accountability) and their extent among students. They also rated effective teaching methods and provided feedback on the introduction of humanism education as a credited course. Data collection occurred via printed, mailed, or e-mailed questionnaires. Descriptive statistics and analysis of variance (ANOVA) were used for data analysis. | All groups selected "morality and a sense of ethics," "sense of accountability," "communication skills," and "empathic ability" as the most important qualities. Professors and students reported that students had a high "sense of accountability" and "morality," but low "empathic ability" and "communication skills." The most preferred teaching methods were extracurricular activities, such as small group activities, debates, and volunteer services. Participants also showed a positive response towards humanism education but were more passive about introducing it as a credited course. A grading method based on participation was favored. | The study concluded that humanism education in medical schools is highly valued by all sub-groups. It is necessary to train faculty and continuously develop new teaching methods to ensure comprehensive and lasting effects of humanism education. A participatory-based grading system was recommended for such courses. | [22] |
| **77** | Enhancing student perspectives of humanism in medicine: Reflections from the Kalaupapa service-learning project. | Qualitative study. | 41 post-baccalaureate students, primarily from disadvantaged backgrounds, including rural communities in Hawai’i and the Pacific. | To determine whether the Kalaupapa service-learning curricula enhanced student perspectives of humanism in medicine at an early stage of their medical training. | Program participants between 2008 and 2014 (n = 41) completed written reflections following the  conclusion of the service-learning project. Four prompts guided student responses. Reflections were thematically  analyzed. Once all essays were read, team members compared their findings to condense or expand themes and  assess levels of agreement. | Emerging themes of resilience and unity were prominent throughout the student reflections. Students expressed respect and empathy for the patients’ struggles and strengths, as well as those of their peers. The experience also reinforced students’ commitment to service, particularly to populations in rural and underserved communities. Students also gained a deeper understanding of the patient experience and of themselves as future physicians. | Training programs must prepare an altruistic health care workforce that embraces the humanistic element of medicine. | [61] |
| **78** | Twelve tips on teaching and learning humanism in medical education. | Literature Review. | Medical students. | To present practical tips for fostering humanistic values in medical education, enhancing the teaching and application of these values across various stages of medical training and professional development. | The authors conducted a literature review, followed by iterative discussions to identify common themes and best practices. The result is a compilation of 12 practical tips for fostering humanistic values, informed by literature and personal experience. | The article identifies 12 tips relevant to contemporary medical education. These include strengthening curricula, incorporating empathy, fostering role modeling, addressing the hidden curriculum, and using assessment tools. | Humanistic values can be effectively incorporated in preclinical and clinical medical education, clinical clerkships, and faculty development. Institutional culture, faculty development, and continuous research are key to sustaining these values. | [20] |
| **79** | A medical student elective promoting humanism, communication skills, complementary and alternative medicine and physician self-care: An evaluation of the heart program. | Cross-sectional observational study. | Fourth-year medical students. | To evaluate the educational impact of the Humanistic Elective in alternative medicine, Activism, and Reflective Transformation (HEART) program and assess whether it met its goals of promoting humanism in medicine, self-care, communication skills, and community building. | The authors conducted a web-based, cross-sectional survey of the first eight cohorts of HEART graduates in 2010. Survey questions assessed respondents’ demographics and perspectives on the educational impact of the elective. Descriptive statistics were used to characterize the sample and qualitative analyses were guided by grounded theory. | Of 168 eligible alumni, 122 (73%) completed the survey. The majority were female (70%), age ≤35 (77%) years, and trained in primary care specialties (66%). Half were attendings in practice. Most respondents felt the elective taught professionalism (89%) and communication skills (92%) well or very well. The majority highly agreed that the elective helped them better cope with stress during residency training (80%), taught them self-care skills (75%), and improved their ability to empathize and connect with patients (71%). Qualitative analysis of the personal and professional impact of the elective identified twelve common themes with self-discovery, self-care, and collegial development/community most frequently cited. | Most HEART graduates endorse learning important skills and benefiting from the experience both personally and professionally. Aspects of the HEART curriculum may help training programs teach professionalism and improve trainee well-being. | [62] |
| **80** | How we train undergraduate medical students in decoding patients' nonverbal clues. | Descriptive Study. | Second-year medical students at the Autonomous University of Barcelona. | The main purpose was to design and implement a training module to enhance medical students' ability to decode patients' nonverbal communication (NVC) and interpret it in a clinical context. | Interactive learning sessions with 4 x 2-hour seminars and 2 x 5-hour visits to a primary care center. Students used observational protocols, video-clips, role-playing, and group discussions. Evaluation was conducted through multiple-choice tests and satisfaction questionnaires. | The key results indicated that students provided positive feedback on the seminars, visits, and the overall program organization. They also demonstrated positive assessment of their ability to decode NVC, especially facial expressions of emotions. Students expressed high satisfaction with the training and educators. | The conclusions emphasized that training in nonverbal communication (NVC) is essential for medical students to improve doctor-patient relationships. It was concluded that NVC training should precede clinical interview training, contributing to students' awareness of psychosocial aspects of care and promoting empathy. | [74] |
| **81** | An investigation of construct validity of humanistic clinical skills on a medical licensure examination. | Cross-sectional observational study. | A random sample of 227 fourth-year medical students from 23  osteopathic medical schools represent the COMLEX-USA Level 2-  PE database of 3754 medical students tested in 2007–2008. Data  include similar numbers of females (51%) and males (50%). The  majority racial/ethnic group was white (78%). | To test the construct validity of the Global Patient Assessment Tool (GPAT) used to assess humanistic clinical skills (professionalism, interpersonal relationships, physician-patient communication) as part of the medical licensure performance examination using multitrait-multimethod confirmatory factor analysis. | The Global Patient Assessment Tool (GPAT), designed to assess professionalism, interpersonal relationship, and doctor–patient communication was tested under two measurement methods: standard examination ratings and peer performance ratings of the same examinee. Two concurrent validity factors (data gathering and written patient notes) were included. | Convergent validity was supported under the two methods of scoring. Moderate to strong correlations among trait factors indicated weak discriminate validity. Method effects were indicated. The relationship between GPAT and two concurrent validity factors indicate measures of relatively different constructs. | Evidence of construct validity for the GPAT indicate scores should be interpreted as measuring a humanistic clinical skills construct consisting of homogeneous measures of professionalism, interpersonal relationship, and physician–patient communication. Findings are consistent with  the interpretation and use of the GPAT as an important part of medical licensure examinations. | [179] |
| **82** | Incorporating professionalism into medical education: The Mayo Clinic experience. | Descriptive Study. | Medical students, physicians in training (residents and fellows). | To explore approaches for teaching and assessing professionalism, with a focus on the Mayo Clinic’s model. | Review of various assessment methods (360-degree reviews, OSCEs, critical incident reports, professionalism portfolios) and how they are applied across different levels of medical training and practice. | Multiple assessment tools are necessary for valid evaluations of professionalism, including peer reviews, faculty assessments, patient feedback, and direct observations. At Mayo Clinic, professionalism is integrated into the culture, education, and assessment programs for all learners and faculty. | Professionalism is a key competency for physicians. Effective teaching and assessment are integral to maintaining high standards in medical practice. The Mayo Clinic’s integrated approach to professionalism fosters improved clinical outcomes and exemplifies its importance as a core competency. | [29] |
| **83** | Long-term outcomes of the new pathway program at Harvard Medical School: A randomized controlled trial. | Randomized Controlled Trial (RCT). | Harvard Medical School students (100 graduates from 1989 and 1990: 50 NP curriculum students and 50 traditional curriculum students). | To evaluate the long-term effects of the New Pathway Program on behaviors and attitudes related to humanistic medicine, lifelong learning, and social learning. | Long-term follow-up study using 1998 telephone interviews with 100 Harvard Medical School graduates (50 from the NP curriculum and 50 from the traditional curriculum). The NP curriculum included problem-based learning, coordinated lectures, labs, and humanistic medicine experiences, while the traditional curriculum focused on basic science lectures and labs. | Out of 22 survey measures, NP and traditional students showed significant differences on five (three related to humanism and two to social learning). Key findings include:  40% of NP students versus 18% of traditional students went on to practice primary care or psychiatry.  NP students rated their preparation for humanistic medicine higher and felt more confident in managing psychosocial issues.  NP students were more likely to believe that early faculty influence continued to shape their thinking.  NP students expressed a greater preference for the pedagogical approaches of their program compared to traditional students.  No significant differences were found between the two groups regarding lifelong learning. | The NP curriculum showed lasting benefits in humanistic medicine, suggesting it can be taught and learned. Differences observed between NP and traditional students persisted from medical school through residency and into practice. | [180] |

**Reference List:**

20. Cohen LG, Sherif YA. Twelve tips on teaching and learning humanism in medical education. Med Teach. 2014;36:680–4. <https://doi.org/10.3109/0142159X.2014.916779>.

22. Jung HY, Kim JW, Lee S, Yoo SH, Jeon JH, Kim T woo, et al. A study of core humanistic competency for developing humanism education for medical students. J Korean Med Sci. 2016;31:829–35. <https://doi.org/10.3346/jkms.2016.31.6.829>

29. Mueller PS. Incorporating Professionalism into Medical Education: The Mayo Clinic Experience. Keio J Med. 2009;58:133–43. https://doi.org/10.2302/kjm.58.133.

34. Vinson AH, Underman K. Clinical empathy as emotional labor in medical work. Soc Sci Med. 2020;251:112904. https://doi.org/10.1016/J.SOCSCIMED.2020.112904.

35. Ditton-Phare P, Loughland C, Duvivier R, Kelly B. Communication skills in the training of psychiatrists: A systematic review of current approaches. Australian and New Zealand Journal of Psychiatry. 2017;51:675–92. https://doi.org/10.1177/0004867417707820/ASSET/IMAGES/LARGE/10.1177_0004867417707820-FIG2.JPEG.

36. Nelson SW, Germann CA, MacVane CZ, Bloch RB, Fallon TS, Strout TD. Intern as Patient: A Patient Experience Simulation to Cultivate Empathy in Emergency Medicine Residents. West J Emerg Med. 2018;19:41–8. https://doi.org/10.5811/WESTJEM.2017.11.35198.

37. Sutherland T, Choi D, Yu C. “Brought to life through imagery” - animated graphic novels to promote empathic, patient-centred care in postgraduate medical learners. BMC Med Educ. 2021;21. https://doi.org/10.1186/S12909-021-02491-4.

38. MOORHEAD R, WINEFIELD H. Teaching Counselling Skills to Fourth-year Medical Students: A Dilemma Concerning Goals. Fam Pract. 1991;8:343–6. https://doi.org/10.1093/fampra/8.4.343.

39. Ghosh SK, Chakraborty S. Assessment of empathy among first-year medical students: A knowledge, attitude and practice study. Italian Journal of Anatomy and Embryology. 2018;123:1–16. https://doi.org/10.13128/IJAE-22983.

40. Montemurro D, Vescovo G, Negrello M, Frigo AC, Cirillo T, Picardi E, et al. Medical professional values and education: A survey on Italian students of the medical doctor school in medicine and surgery. N Am J Med Sci. 2013;5:134–9. https://doi.org/10.4103/1947-2714.107535.

41. Bayne H, Neukrug E, Hays D, Britton B. A comprehensive model for optimizing empathy in person-centered care. Patient Educ Couns. 2013;93:209–15. https://doi.org/10.1016/j.pec.2013.05.016.

43. Sevrain-Goideau M, Gohier B, Bellanger W, Annweiler C, Campone M, Coutant R. Forum theater staging of difficult encounters with patients to increase empathy in students: Evaluation of efficacy at the University of Angers Medical School. BMC Med Educ. 2020;20:58. https://doi.org/10.1186/s12909-020-1965-4.

44. Stepien KA, Baernstein A. Educating for empathy: A review. J Gen Intern Med. 2006;21:524–30. https://doi.org/10.1111/j.1525-1497.2006.00443.x.

45. Garden R. Expanding clinical empathy: An activist perspective. J Gen Intern Med. 2009;24:122–5. <https://doi.org/10.1007/s11606-008-0849-9>.

46. Fernández-Olano C, Montoya-Fernandez J, Salinas-Sánchez A. Impact of clinical interview training on the empathy level of medical students and medical residents. Med Teach. 2008;30:322–4. https://doi.org/10.1080/01421590701802299.

47. Sullivan C, Ellison SR, Quaintance J, Arnold L, Godrey P. Development of a Communication Curriculum for Emergency Medicine Residents. Teach Learn Med. 2009;21:327–33. https://doi.org/10.1080/10401330903228604.

48. Sulzer SH, Feinstein NW, Wendland CL. Assessing empathy development in medical education: a systematic review. Med Educ. 2016;50:300–10. https://doi.org/10.1111/medu.12806.

49. Pedersen R. Empirical research on empathy in medicine—A critical review. Patient Educ Couns. 2009;76:307–22. https://doi.org/10.1016/J.PEC.2009.06.012.

50. Evans BJ, Stanley RO, Burrows GD. Measuring medical students’ empathy skills. Br J Med Psychol. 1993;66 ( Pt 2):121–33. https://doi.org/10.1111/J.2044-8341.1993.TB01735.X.

51. Dereboy C, Harlak H, Gürel S, Gemalmaz A, Eskin M. Teaching empathy in medical education. Turk Psikiyatri Derg. 2005;16:83–9.

52. Ye X, Guo H, Xu Z, Xiao H. Empathy variation of undergraduate medical students after early clinical contact: a cross-sectional study in China. BMJ Open. 2020;10. https://doi.org/10.1136/BMJOPEN-2019-035690.

53. Tidhar M, Benbassat J. Teaching Shared Decision Making to Undergraduate Medical Students. Rambam Maimonides Med J. 2021;12. https://doi.org/10.5041/RMMJ.10453.

54. Kagawa Y, Ishikawa H, Son D, Okuhara T, Okada H, Ueno H, et al. Using patient storytelling to improve medical students’ empathy in Japan: a pre-post study. BMC Med Educ. 2023;23:67. https://doi.org/10.1186/s12909-023-04054-1.

55. Yuan J, Zeng X, Cheng Y, Lan H, Cao K, Xiao S. Narrative medicine in clinical internship teaching practice. Med Educ Online. 2023;28:2258000. https://doi.org/10.1080/10872981.2023.2258000.

56. Denizon Arranz S, Monge Martín D, Caballero Martínez F, Neria Serrano F, Chica Martínez P, Ruiz Moral R. A Multifaceted Educational Intervention in the Doctor–Patient Relationship for Medical Students to Incorporate Patient Agendas in Simulated Encounters. Healthcare. 2023;11:1699. https://doi.org/10.3390/healthcare11121699.

57. Doukas DJ, Ozar DT, Darragh M, de Groot JM, Carter BS, Stout N. Virtue and care ethics & humanism in medical education: a scoping review. BMC Med Educ. 2022;22:131. https://doi.org/10.1186/s12909-021-03051-6.

58. Hazrati H, Bigdeli S, Gavgani VZ, Soltani Arabshahi SK, Behshid M, Sohrabi Z. Humanism in clinical education: A mixed methods study on the experiences of clinical instructors in Iran. Philosophy, Ethics, and Humanities in Medicine. 2020;15:5. https://doi.org/10.1186/s13010-020-00088-1.

59. Canales C, Strom S, Anderson CT, Fortier MA, Cannesson M, Rinehart JB, et al. Humanistic medicine in anaesthesiology: development and assessment of a curriculum in humanism for postgraduate anaesthesiology trainees. Br J Anaesth. 2019;123:887–97. https://doi.org/10.1016/j.bja.2019.08.021.

60. Montgomery L, Loue S, Stange KC. Linking the heart and the head: Humanism and professionalism in medical education and practice. Fam Med. 2017;49:378–83.

61. Lee WK, Harris CCD, Mortensen KA, Long LM, Sugimoto-Matsuda J. Enhancing student perspectives of humanism in medicine: Reflections from the Kalaupapa service learning project. BMC Med Educ. 2016;16:137. https://doi.org/10.1186/s12909-016-0664-7.

62. Dossett ML, Kohatsu W, Nunley W, Mehta D, Davis RB, Phillips RS, et al. A medical student elective promoting humanism, communication skills, complementary and alternative medicine and physician self-care: An evaluation of the heart program. Explore: The Journal of Science and Healing. 2013;9:292–8. https://doi.org/10.1016/j.explore.2013.06.003.

63. Lorenz FJ, Darok MC, Ho L, Holstrom-Mercader MS, Freiberg AS, Dellasega CA. The Impact of an Unconventional Elective in Narrative Medicine and Pediatric Psycho-oncology on Humanism in Medical Students. Journal of Cancer Education. 2022;37:1798–805. https://doi.org/10.1007/s13187-021-02029-8.

64. Schaff PB, Isken S, Tager RM. From contemporary art to core clinical skills: observation, interpretation, and meaning-making in a complex environment. Acad Med. 2011;86:1272–6. https://doi.org/10.1097/ACM.0B013E31822C161D.

65. Ju M, Berman AT, Hwang W-T, LaMarra D, Baffic C, Suneja G, et al. Assessing Interpersonal and Communication Skills in Radiation Oncology Residents: A Pilot Standardized Patient Program. Int J Radiat Oncol Biol Phys. 2014;88:1129–35. https://doi.org/10.1016/j.ijrobp.2014.01.007.

66. Epner DE, Reddy SK, Hui D, Fellman B, Bruera E. Doing the hard work of learning: oncologists’ enduring impressions of a year-long communication skills training program. Supportive Care in Cancer. 2024;32:71. https://doi.org/10.1007/s00520-023-08285-2.

67. Yudkowsky R, Downing SM, Ommert D. Prior experiences associated with residents’ scores on a communication and interpersonal skill OSCE. Patient Educ Couns. 2006;62:368–73. https://doi.org/10.1016/j.pec.2006.03.004.

68. Fallowfield L, Jenkins V, Farewell V, Solis-Trapala I. Enduring impact of communication skills training: results of a 12-month follow-up. Br J Cancer. 2003;89:1445–9. https://doi.org/10.1038/sj.bjc.6601309.

69. Larkin AC, Cahan MA, Whalen G, Hatem D, Starr S, Haley HL, et al. Human emotion and response in surgery (HEARS): A simulation-based curriculum for communication skills, systems-based practice, and professionalism in surgical residency training. J Am Coll Surg. 2010;211:285–92. https://doi.org/10.1016/j.jamcollsurg.2010.04.004.

70. Rivet EB, Edwards C, Lange P, Haynes S, Feldman M, Cholyway R. Telehealth Training for Surgeons to Empathetically Deliver Bad News Via Video-Mediated Communication. American Surgeon. 2023;89:440–6. https://doi.org/10.1177/00031348211030458.

71. Vegni E, Moja EA. Effects of a course on ophthalmologist communication skills: A pilot study. Education for Health. 2004;17:163–71. https://doi.org/10.1080/13576280410001710987.

72. Carvalho IP, Pais VG, Silva FR, Martins R, Figueiredo-Braga M, Pedrosa R, et al. Teaching communication skills in clinical settings: comparing two applications of a comprehensive program with standardized and real patients. BMC Med Educ. 2014;14:92. https://doi.org/10.1186/1472-6920-14-92.

73. Courteille O, Josephson A, Larsson L-O. Interpersonal behaviors and socioemotional interaction of medical students in a virtual clinical encounter. BMC Med Educ. 2014;14:64. https://doi.org/10.1186/1472-6920-14-64.

74. Molinuevo B, Escorihuela RM, Fernndez-Teruel A, Tobeña A, Torrubia R. How we train undergraduate medical students in decoding patients’ nonverbal clues. Med Teach. 2011;33:804–7. https://doi.org/10.3109/0142159X.2011.588737.

75. Arntfield SL, Slesar K, Dickson J, Charon R. Narrative medicine as a means of training medical students toward residency competencies. Patient Educ Couns. 2013;91:280–6. https://doi.org/10.1016/j.pec.2013.01.014.

76. Lijoi AF, Tovar AD. Narrative medicine: Re-engaging and re-energizing ourselves through story. Int J Psychiatry Med. 2020;55:321–30. https://doi.org/10.1177/0091217420951039.

77. Huang C Da, Jenq CC, Liao KC, Lii SC, Huang CH, Wang TY. How does narrative medicine impact medical trainees’ learning of professionalism? A qualitative study. BMC Med Educ. 2021;21:1–8. https://doi.org/10.1186/S12909-021-02823-4/PEER-REVIEW.

79. Lawrence K, Cho J, Torres C, Alfaro-arias V. Building Virtual Health Training Tools for Residents: A Design Thinking Approach. Front Digit Health. 2022;4:861579. https://doi.org/10.3389/fdgth.2022.861579.

78. Leijenaar E, Eijkelboom C, Milota M. “An invitation to think differently”: a narrative medicine intervention using books and films to stimulate medical students’ reflection and patient-centeredness. BMC Med Educ. 2023;23:568. https://doi.org/10.1186/s12909-023-04492-x.

80. Jeppu AK, Kumar KA, Sethi A. ‘We work together as a group’: implications of jigsaw cooperative learning. BMC Med Educ. 2023;23:734. https://doi.org/10.1186/s12909-023-04734-y.

81. Franco RS, Dos Santos Franco CAG, Karnieli-Miller O. Deepening the teaching and learning of clinical communication: The importance of reflection and feedback in health education[Aprofundando o ensino e aprendizagem de comunicação clínica: A importância da reflexão e do feedback no ensino em saúde]. Sci Med (Porto Alegre). 2021;31:8. https://doi.org/10.15448/1980-6108.2021.1.39795.

82. Lane AS, Roberts C. Developing open disclosure strategies to medical error using simulation in final-year medical students: linking mindset and experiential learning to lifelong reflective practice. BMJ Simul Technol Enhanc Learn. 2020;:bmjstel-2020-000659. https://doi.org/10.1136/bmjstel-2020-000659.

83. Gilligan C, Brubacher SP, Powell MB. “We’re All Time Poor”: Experienced Clinicians’ and Students’ Perceptions of Challenges Related to Patient Communication. Teach Learn Med. 2022;34:1–12. https://doi.org/10.1080/10401334.2021.1893175.

84. Orgel E, McCarter R, Jacobs S. A Failing Medical Educational Model: A Self-Assessment by Physicians at All Levels of Training of Ability and Comfort to Deliver Bad News. J Palliat Med. 2010;13:677–83. https://doi.org/10.1089/jpm.2009.0338.

85. Udyavar R, Smink DS, Mullen JT, Kent TS, Green A, Harlow AF, et al. Qualitative Analysis of a Cultural Dexterity Program for Surgeons: Feasible, Impactful, and Necessary. J Surg Educ. 2018;75:1159–70. https://doi.org/10.1016/j.jsurg.2018.01.016.

86. Archer E, Meyer IS. Applying empathic communication skills in clinical practice: Medical students’ experiences. South African Family Practice. 2021;63:1–5. https://doi.org/10.4102/safp.v63i1.5244.

87. Brouwers MH, Bor H, Laan R, van Weel C, van Weel-Baumgarten E. Students’ experiences with a longitudinal skills training program on breaking bad news: A follow-up study. Patient Educ Couns. 2018;101:1639–44. https://doi.org/10.1016/j.pec.2018.05.008.

88. Lawrence K, Hanley K, Adams J, Sartori DJ, Greene R, Zabar S. Building Telemedicine Capacity for Trainees During the Novel Coronavirus Outbreak: a Case Study and Lessons Learned. J Gen Intern Med. 2020;35:2675–9. https://doi.org/10.1007/s11606-020-05979-9.

92. Duffy FD, Gordon GH, Whelan G, Cole-Kelly K, Frankel R. Assessing competence in communication and interpersonal skills: The Kalamazoo II report. Academic Medicine. 2004;79:495–507. https://doi.org/10.1097/00001888-200406000-00002.

93. Kamath L, Chakraborty A, Asanaliyar M. Perception and reflections of second year medical students on attitude, ethics, and communication skills module on patient autonomy and decision making in a tertiary care teaching hospital. JMS - Journal of Medical Society. 2023;37:51–6. https://doi.org/10.4103/jms.jms_86_22.

100. Menezes P, Guraya SY, Guraya SS. A Systematic Review of Educational Interventions and Their Impact on Empathy and Compassion of Undergraduate Medical Students. Front Med (Lausanne). 2021;8:758377. https://doi.org/10.3389/fmed.2021.758377.

108. Arora S, Ashrafian H, Davis R, Athanasiou T, Darzi A, Sevdalis N. Emotional intelligence in medicine: a systematic review through the context of the ACGME competencies. Med Educ. 2010;44:749–64. https://doi.org/10.1111/j.1365-2923.2010.03709.x.

110. Hammerly ME, Harmon L, Schwaitzberg SD. Good to Great: Using 360-Degree Feedback to Improve Physician Emotional Intelligence. Journal of Healthcare Management. 2014;59:354–66. https://doi.org/10.1097/00115514-201409000-00009.

118. Lehmann LS, Sulmasy LS, Desai S. Hidden curricula, ethics, and professionalism: Optimizing clinical learning environments in becoming and being a physician: A position paper of the American college of physicians. Ann Intern Med. 2018;168:506–8. https://doi.org/10.7326/M17-2058.

146. Meyer GS, Potter A, Gary N. A national survey to define a new core curriculum to prepare physicians for managed care practice. Academic Medicine. 1997;72:669–76. https://doi.org/10.1097/00001888-199708000-00010.

147. Stratton TD, Elam CL, Murphy-Spencer AE, Quinlivan SL. Emotional Intelligence and Clinical Skills: Preliminary Results from a Comprehensive Clinical Performance Examination. Academic Medicine. 2005;80 Supplement:S34–7. https://doi.org/10.1097/00001888-200510001-00012.

152. Browning DM, Meyer EC, Truog RD, Solomon MZ. Difficult conversations in health care: Cultivating relational learning to address the hidden curriculum. Academic Medicine. 2007;82:905–13. <https://doi.org/10.1097/ACM.0b013e31812f77b9>.

155. Feng X, Wang Y, Wei L, Meng K. How to become an excellent pediatric resident: a qualitative comparative study from China. BMC Health Serv Res. 2023;23:53. https://doi.org/10.1186/s12913-023-09038-x.

157. Chessman AW, Blue A V., Gilbert GE, Carey M, Mainous AG. Assessing students’ communication and interpersonal skills across evaluation settings. Fam Med. 2003;35:643–8.

168. Luff D, Martin EB, Mills K, Mazzola NM, Bell SK, Meyer EC. Clinicians’ strategies for managing their emotions during difficult healthcare conversations. Patient Educ Couns. 2016;99:1461–6. https://doi.org/10.1016/j.pec.2016.06.017.

169. Adeyemi O, Ginsburg AD, Kaur R, Cuthel AM, Zhao N, Siman N, et al. Serious illness communication skills training for emergency physicians and advanced practice providers: a multi-method assessment of the reach and effectiveness of the intervention. BMC Palliat Care. 2024;23:48. https://doi.org/10.1186/s12904-024-01349-y.

170. Palimar V, Gupta C, Rao P. Undergraduate students perspective on ethics and communication modules. J Punjab Acad Forensic Med Toxicol. 2023;23:186–9. https://doi.org/10.5958/0974-083X.2023.00029.8.

171. Vern-Gross TZ, Laughlin BS, Kough K, Ernst B, Langley N, Rule WG, et al. Implementation of the REFLECT Communication Curriculum for Clinical Oncology Graduate Medical Education. J Palliat Med. 2024;27:231–5. https://doi.org/10.1089/jpm.2023.0316.

172. Atasoy BM, Sarikaya O, Kuscu MK, Yondem M, Buyukkara E, Eken EG, et al. Students Meeting with Caregivers of Cancer Patient: Results of an Experience-Based Learning Project. Journal of Cancer Education. 2012;27:656–63. https://doi.org/10.1007/s13187-012-0387-3.

173. de Moura Villela EF, Bastos LK, de Almeida WS, Pereira AO, de Paula Rocha MS, de Oliveira FM, et al. Effects on Medical Students of Longitudinal Small-Group Learning about Breaking Bad News. Perm J. 2020;24:157. https://doi.org/10.7812/TPP/19.157.

174. Porter AS, Woods C, Kaye EC, Aglio T, Applegarth J, Bien K, et al. Novel approaches to communication skills development: The untapped potential of qualitative research immersion. PEC Innovation. 2022;1:100079. https://doi.org/10.1016/j.pecinn.2022.100079.

175. Spencer J, Blackmore D, Heard S, McCrorie P, McHaffie D, Scherpbier A, et al. Patient-oriented learning: A review of the role of the patient in the education of medical students. Med Educ. 2000;34:851–7. https://doi.org/10.1046/j.1365-2923.2000.00779.x.

176. Lombardo L, Ehlers J, Lutz G. Mindset and Reflection—How to Sustainably Improve Intra- and Interpersonal Competences in Medical Education. Healthcare (Switzerland). 2023;11. https://doi.org/10.3390/HEALTHCARE11060859/S1.

177. Buck E, Billingsley T, McKee J, Richardson G, Geary C. The physician healer track: educating the hearts and the minds of future physicians. Med Educ Online. 2021;26:1844394. https://doi.org/10.1080/10872981.2020.1844394.

178. Mustika R, Soemantri D. Unveiling the hurdles in cultivating humanistic physicians in the clinical setting: An exploratory study. Malaysian Journal of Medical Sciences. 2020;27:117–24. https://doi.org/10.21315/mjms2020.27.3.12.

179. Roberts WL, Solomon M, Langenau E. An investigation of construct validity of humanistic clinical skills on a medical licensure examination. Patient Educ Couns. 2011;82:214–21. https://doi.org/10.1016/j.pec.2010.03.016.

180. Peters AS, Greenberger-Rosovsky R, Crowder C, Block SD, Moore GT. Long-term outcomes of the new pathway program at Harvard Medical School: A randomized controlled trial. Academic Medicine. 2000;75:470–9. <https://doi.org/10.1097/00001888-200005000-00018>.

181. Jouriles NJ, Emerman CL, Cydulka RK. Direct Observation for Assessing Emergency Medicine Core Competencies: Interpersonal Skills. Academic Emergency Medicine. 2002;9:1338–41. https://doi.org/10.1111/j.1553-2712.2002.tb01598.x.

182. Suciu N, Meliț LE, Mărginean CO. A Holistic Approach of Personality Traits in Medical Students: An Integrative Review. Int J Environ Res Public Health. 2021;18:12822. https://doi.org/10.3390/ijerph182312822.
